# A mast cell receptor mediates post-stroke brain inflammation via a dural-brain axis

**DOI:** 10.1016/j.cell.2025.06.045

**Published:** 2025-07-24

**Authors:** Ruchita Kothari, Mostafa W. Abdulrahim, Hyun Jong Oh, Daniel H. Capuzzi, Collin B. Kilgore, Sumil K. Nair, Yaowu Zhang, Nathachit Limjunyawong, Sarbjit S. Saini, Jennifer E. Kim, Justin M. Caplan, Fernanado L. Gonzalez, Christopher M. Jackson, Chetan Bettegowda, Judy Huang, Bhanu P. Ganesh, Chunfeng Tan, Raymond C. Koehler, Rafael J. Tamargo, Louise D. McCullough, Risheng Xu, Xinzhong Dong

**Affiliations:** 1 The Solomon H. Snyder Department of Neuroscience, Johns Hopkins University School of Medicine, Baltimore, MD 21205; 2 Department of Neurosurgery, Johns Hopkins University School of Medicine, Baltimore, MD 21205; 3 Johns Hopkins Asthma and Allergy Center, Baltimore, MD 21224; 4 Department of Neurosurgery, The Ohio State University College of Medicine, Columbus, OH 43210; 5 Department of Neurology, The University of Texas Health Science Center Houston, McGovern Medical School, Houston, TX 77030; 6 Department of Anesthesiology and Critical Care Medicine, Johns Hopkins University School of Medicine, Baltimore, MD 21205; 7 Department of Dermatology, Johns Hopkins University School of Medicine, Baltimore, MD 21205; 8 Howard Hughes Medical Institute, Chevy Chase, MD 20815; 9 These authors contributed equally; 11 Lead contact

**Keywords:** Mrgpr receptor, mast cell, substance P, ischemic stroke, meninges, inflammation, skull bone marrow, semaphorin

## Abstract

The immune environment surrounding the brain plays a fundamental role in monitoring signs of injury. Insults, including ischemic stroke, can disrupt this balance and incite an exaggerated inflammatory response, yet the underlying mechanism remains unclear. Here, we show that the mast cell-specific receptor Mrgprb2 regulates post-stroke brain inflammation from the meninges. Mrgprb2 causes meningeal mast cell degranulation after stroke, releasing immune mediators. This recruits skull bone marrow neutrophils into the dura and further promotes neutrophil migration from the dura into the brain by cleaving the chemorepellent semaphorin3a. We demonstrate that the human ortholog, MRGPRX2, is expressed in human meningeal mast cells, and is activated by upregulation of the neuropeptide substance P in stroke. Pharmacologically inhibiting Mrgprb2 reduces post-stroke inflammation and improves neurological outcomes in mice, providing a druggable target. Collectively, our study identifies Mrgprb2 as a critical meningeal gatekeeper for immune migration from skull bone marrow reservoirs into the brain.

## In Brief

Meningeal mast cells, via the receptor Mrgprb2/X2, orchestrate brain inflammation after stroke by recruiting and promoting the transfer of skull bone marrow neutrophils into the brain, highlighting these mast cells as key regulators of brain immunity and of the skull bone marrow-meningeal-brain network.

## Graphical Abstract

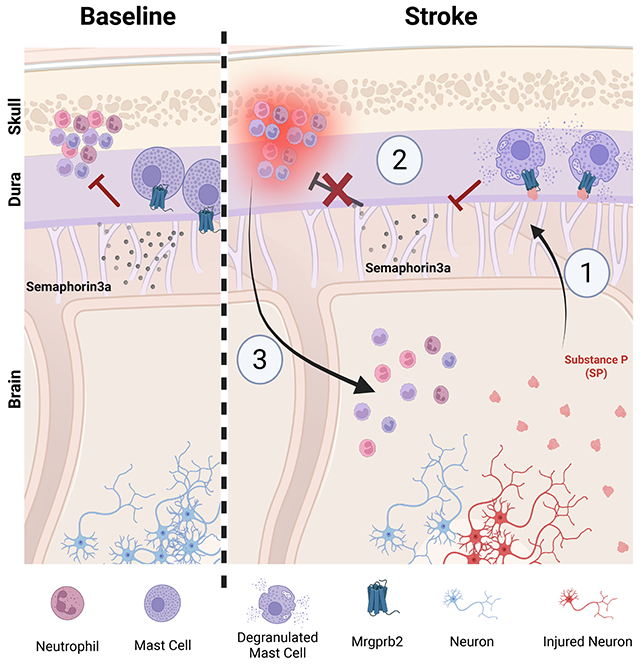

## INTRODUCTION

Immune surveillance is tightly regulated within the central nervous system (CNS) through resident and peripheral immune cells that strategically monitor the brain for signs of injury. This physiologic relationship is key to maintaining homeostasis but can become pathologic following injuries such as stroke. While inflammation after ischemic stroke can have a dual beneficial and detrimental effect, infiltration of neutrophils and monocytes^1^ into the brain has been correlated with greater infarct volume and long-term functional deficits months to years after stroke^2–6^. This acute inflammation has also been shown to permanently alter the brain’s immune environment^7–9^, and thus, understanding specific mechanisms that mediate brain inflammation after injury is vital.

A growing body of evidence suggests that the meninges surrounding the CNS plays a central role in immunosurveillance^10–12^. Indeed, the discovery of meningeal lymphatics that drain the CNS questioned the concept that the brain is an immune-privileged site and has launched further studies into how the meninges play a role in brain immunity^13,14^. However, how meningeal immune cells respond to ischemic stroke is unclear and specific molecules that sense and mediate immune recruitment are unknown^15–18^.

One such cell that has been previously studied but often overlooked is the mast cell. Better known for their role in allergic diseases, mast cells have more recently been identified as primary immune responders in several models of neuronal injury^19,20^. Through the discovery of murine Mrgprb2 and human MRGPRX2^21^, mast cell-specific members of the Mas-related family of G-protein-coupled receptors (Mrgprs), the role of mast cells as sentinel immune cells responsible for probing the local environment and propagating inflammation has become evident. Mrgprb2/X2 are activated by key neuroimmune signaling molecules, including the neuropeptides substance P and pituitary adenylate cyclase-activating polypeptide^22–27^. Despite an extensive body of work describing the roles of Mrgprb2/X2 in peripheral inflammation, whether these receptors contribute to inflammation in the brain remains unclear.

Here, we demonstrate that Mrgprb2/X2 are key receptors necessary for mast cell activation after ischemic stroke in mice and humans. Mouse meningeal mast cells sense neuronal injury in the brain after stroke and release cytokines to recruit skull bone marrow neutrophils into the brain. Specifically, Mrgprb2-mediated mast cell activity suppresses the chemorepellent barrier between the dura and brain, allowing neutrophils to translocate into regions of cerebral ischemia. Further, genetic deletion and pharmacological inhibition of Mrgprb2 attenuates post-stroke inflammation and decreases infarct volume, reducing mortality and improving functional outcomes in mice. Our findings identify the Mrgprb2 receptor as a regulatory component within the meninges that bridges skull bone marrow neutrophil reservoirs to the brain following stroke. Inhibiting Mrgprb2/X2 in the early phase of ischemic stroke may therefore be a promising therapeutic intervention to attenuate the immune response and improve neurologic outcomes.

## RESULTS

### Mrgprb2 contributes to post ischemic stroke injury.

**Figure 1.**
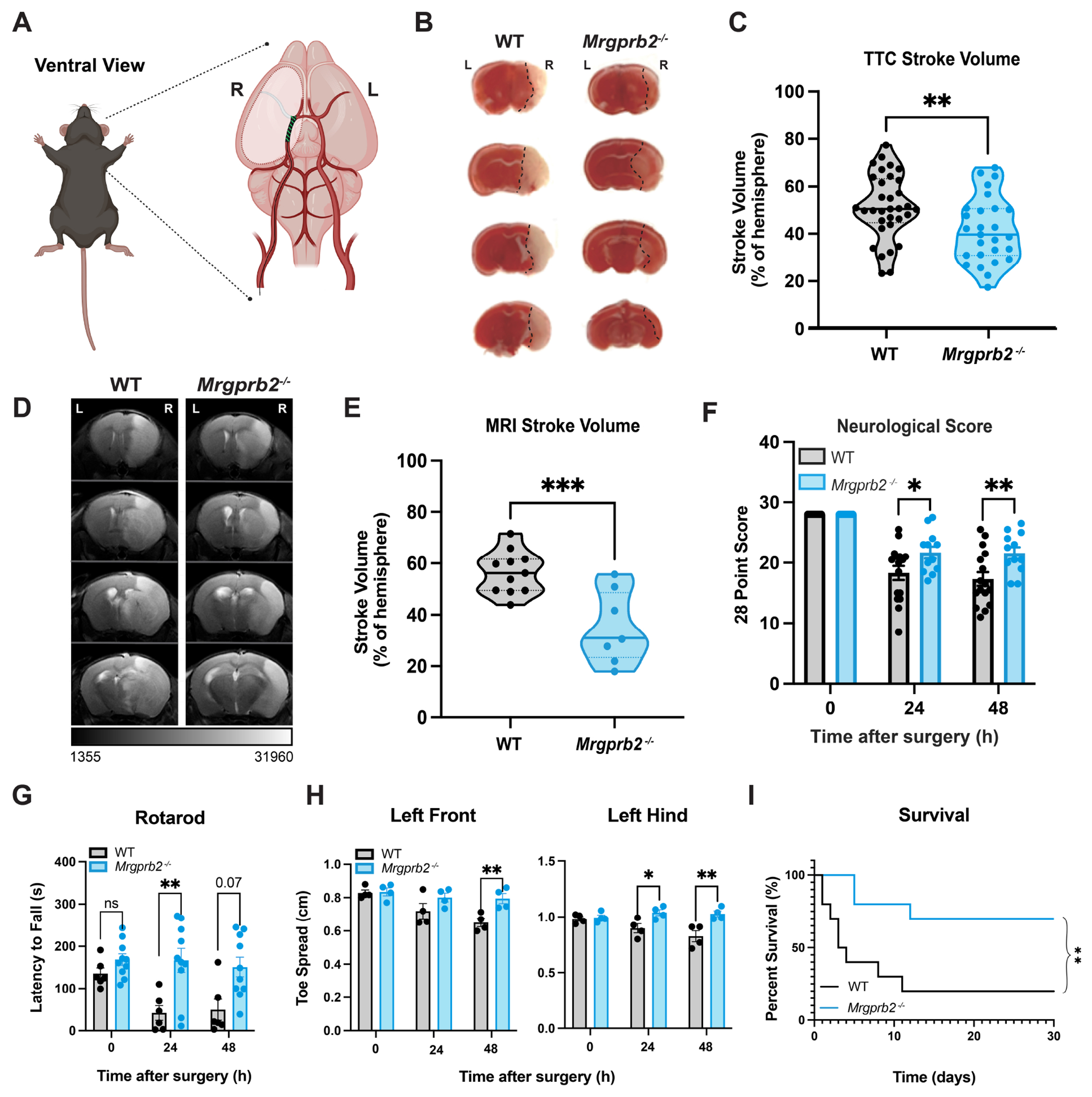
*Mrgprb2*^*−/−*^ mice are protected from ischemic stroke injury. (A) Schematic of transient middle cerebral artery occlusion (tMCAO) model. Black and green dotted line indicates filament and white vessel indicates MCA that is occluded (BioRender). (B) Representative 2,3,5-triphenyltetrazolium (TTC) staining of WT/*Mrgprb2*^*−/−*^ mice brains. Each column represents one brain from anterior to posterior. Infarct is denoted by region of brain with no red dye uptake, separated by dotted line. (C) Quantification of WT/*Mrgprb2*^*−/−*^ brain stroke volumes using TTC stain 48h post-tMCAO (WT *n*=33, *Mrgprb2*^*−/−*^ *n*=27). (D) Representative T2-weighted MRIs of brains of WT/*Mrgprb2*^*−/−*^ mice 48h post-tMCAO. Each column represents one brain from anterior to posterior. Infarct is denoted by brighter signal in the right hemisphere. Signal intensity (in arbitrary units) is denoted by color bar. (E) Quantification of stroke volume in WT/*Mrgprb2*^*−/−*^ mice 48h post-tMCAO (WT *n*=11, *Mrgprb2*^*−/−*^ *n*=8). (F) WT/*Mrgprb2*^*−/−*^ mice neurological scores pre- and post-tMCAO. Higher score indicates better overall sensorimotor function (WT *n*=16, *Mrgprb2*^*−/−*^ *n*=12). (G) Latency to fall on rotarod for WT/*Mrgprb2*^*−/−*^ mice pre- and post-tMCAO. Higher latency to fall indicates better motor function (WT *n*=6, *Mrgprb2*^*−/−*^ *n*=10). (H) *left*, Left front paw and *right,* left hind paw toe spread pre- and post-tMCAO. (WT *n*=4, *Mrgprb2*^*−/−*^ *n*=4). (I) Kaplan-Meier survival curve of WT/*Mrgprb2*^*−/−*^ mice post-tMCAO. (WT *n*=10, *Mrgprb2*^*−/−*^ *n*=10). Statistical analyses: two-sided Student’s t-test (C and E), two-way ANOVA with Sidak’s multiple comparisons test (F and H), Kruskal-Wallis test (G), and log-rank test (I). Bar graphs indicate mean ± SEM. ns, not significant; *P < 0.05, **P < 0.01, ***P < 0.001.

We first sought to investigate whether mast cells contribute to ischemic stroke pathology via Mrgprb2. To simulate ischemic stroke, we performed transient middle cerebral artery occlusion (tMCAO)^28,29^ in wild-type (WT) and Mrgprb2-null (*Mrgprb2^−/−^*) mice (Figure 1A). We observe no difference in blood flow reduction via laser doppler during the tMCAO surgery between WT and *Mrgprb2^−/−^* mice (Figure S1A), indicating equivalent ischemic stroke induction. After 40 minutes, the filament was removed, and the mice were placed in recovery for 48 hours before measuring the volume of infarcted brain tissue. With the chemical stain 2,3,5-triphenyltetrazolium chloride (TTC) that stains live but not infarcted tissue, we find that *Mrgprb2^−/−^* mice exhibit reduced stroke volume (Figure 1B–C). We confirmed this finding with MRI and see that *Mrgprb2^−/−^* mice show a 22% ± 5% reduction in stroke volume as compared to WT mice at 48 hours (Figure 1D–E). *Mrgprb2^−/−^* mice also have reduced midline shift, consistent with less edema and parenchymal injury (Figure S1B).

To delineate whether these quantifiable differences in infarct volume translate to differences in focal neurological deficits, we employed several behavior tests^30^. Using the 28-point neuroscore, we find that WT mice suffer from worse sensorimotor deficits as early as 24 hours after tMCAO when compared to *Mrgprb2^−/−^* mice (Figure 1F). WT mice also perform worse in the rotarod test compared to *Mrgprb2^−/−^* mice, demonstrating severe motor deficits (Figure 1G). Consistent with an infarct of the right temporal hemisphere, WT mice also experience a diminished left front and hind paw spread (distance between the first and fourth toes) by Catwalk (Figure 1H), which is not seen in the right paws (Figure S1C). In addition to fewer functional neurologic deficits, *Mrgprb2^−/−^* mice show increased survival 30 days after tMCAO (Figure1I).

### Mrgprb2-expressing meningeal mast cells are activated after stroke.

**Figure 2.**
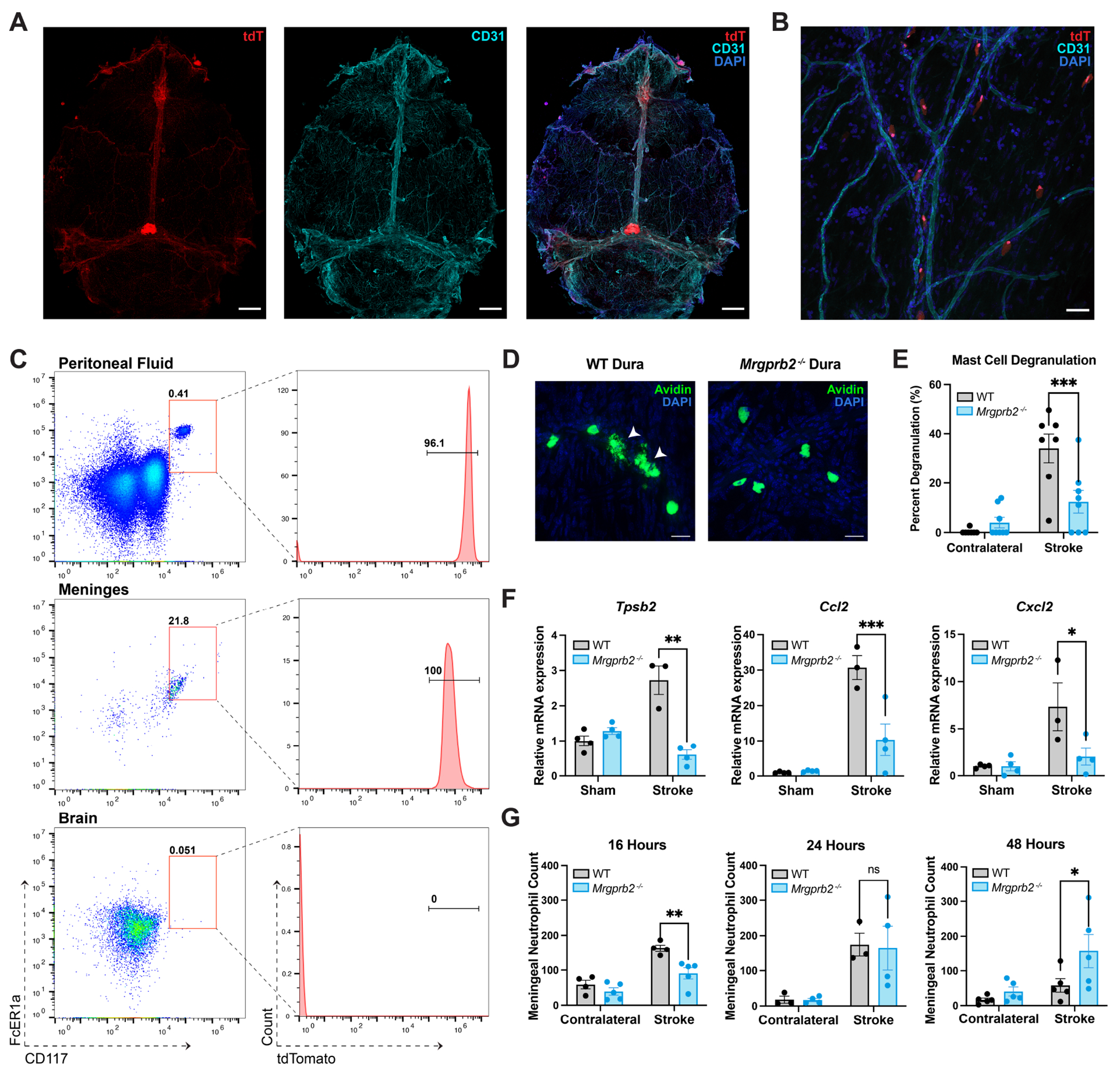
Mrgprb2 is expressed in meningeal mast cells and is activated after tMCAO. (A) Whole mount dura from Mrgprb2-Cre;tdT mice. *left,* tdT signal correlates to Mrgprb2 expression. *middle,* CD31 counterstain identifies vasculature. *right,* Merged channels with DAPI. Scale bar=500 μm. (B) Magnified whole mount dura. Scale bar=25 μm. (C) Flow cytometry of Mrgprb2-Cre;tdT peritoneal fluid, dura, and whole brain. *left,* Live CD45-positive cells gated for mast cells using CD117 and FcER1α. Number is frequency of mast cells among CD45-positive cells. *right*, Mast cells gated for tdT fluorescence represented as cell count versus intensity. Number is percentage of mast cells that are tdT-positive. (D) Representative immunofluorescence images of WT/*Mrgprb2*^*−/−*^ right hemispheric dura 6h post-tMCAO. Avidin denotes mast cell stain and DAPI identifies nuclei. White arrows indicate degranulated cells. Scale bar=25 μm. (E) Percent of degranulated mast cells in WT/*Mrgprb2*^*−/−*^ meninges in dura overlaying the contralateral/stroke brain hemispheres (WT *n*=7, *Mrgprb2*^*−/−*^ *n*=8). (F) Relative mRNA expression in WT/*Mrgprb2*^*−/−*^ dura 48h post-tMCAO, normalized to WT sham expression (WT sham *n*=4, *Mrgprb2*^*−/−*^ sham *n*=4, WT MCAO *n*=3, *Mrgprb2*^*−/−*^ MCAO *n*=4). (G) Neutrophil count/frame in WT/*Mrgprb2*^*−/−*^ dura overlaying the contralateral/stroke brain hemisphere post-tMCAO. (*left*, WT *n*=4, *Mrgprb2*^*−/−*^ *n*=5, *middle*, WT *n*=3, *Mrgprb2*^*−/−*^ *n*=4, *right*, WT *n*=5, *Mrgprb2*^*−/−*^ *n*=5). Statistical analyses: two-way ANOVA with Sidak’s multiple comparisons test (E-G), and Kruskal-Wallis test (F, Tpsb2). Bar graphs indicate mean ± SEM. ns, not significant; *P < 0.05, **P < 0.01, ***P < 0.001.

We next wanted to delineate the location of these Mrgprb2-expressing mast cells in the context of ischemic stroke. To elucidate whether Mrgprb2 mast cells are present in the meninges^31^ or brain, we leveraged Mrgprb2-Cre;Rosa26-tdTomato (tdT) reporter mice which express tdT only in cells expressing Mrgprb2^21^. We observe Mrgprb2 expression in dural mast cells and find that these cells are in close proximity to the vasculature, as seen by CD31 staining (Figure 2A–B). Using the common mast cell marker avidin, we detect tdT in all dural mast cells, thus confirming that 100% of dural mast cells express Mrgprb2 (Figure S2A–B).

Flow cytometric analyses confirm that these tdT-positive cells are CD117- and FcER1α- positive mast cells and demonstrate that Mrgprb2-expressing mast cells are restricted to the dura and are not present in the brain parenchyma (Figure 2C, Figure S2C–E). We additionally examined whether these mast cells migrate into the brain after stroke, but do not observe any Mrgprb2-expressing mast cells in the brain 48 hours after tMCAO (Figure S2F–G) nor any change in the number of mast cells in the dura (Figure S2H). These observations are corroborated by three independent studies of the single cell transcriptome of the whole mouse brain at baseline^32,33^ (Figure S2I) and after ischemic stroke^34^, all showing no Mrgprb2 expression in the brain. These data suggest that Mrgprb2-mast cells remain in their native dural compartment and incite pathologic brain inflammation from the meninges.

To measure the activity of these dural mast cells, we monitored the release of small vesicles near mast cells as a marker of degranulation. We first show that WT and *Mrgprb2^−/−^* meningeal mast cells exhibit very low activation after sham surgery and show no differences in degranulation between dural hemispheres (Figure S2J). We find that deleting Mrgprb2 significantly decreases mast cell activation after ischemic stroke, as WT dural mast cells in the dura overlaying the stroke hemisphere are significantly more degranulated after tMCAO than *Mrgprb2^−/−^* cells (Figure 2D–E). We also see an increase in expression of the mast cell-specific serine protease tryptase beta 2 (Tpsb2), in WT over *Mrgprb2^−/−^* meninges following tMCAO, consistent with increased mast cell activation in WT meninges (Figure 2F). We additionally assessed other mast cells factors^35^ as biomarkers of increased inflammatory burden in WT meninges (Figure S2K). Although not specific to mast cells, CCL2 and CXCL2, chemokines involved in monocyte and neutrophil recruitment, respectively, are upregulated in the meninges of WT mice. These findings suggest a role of Mrgprb2 in promoting general inflammation in the meninges after tMCAO. Proinflammatory markers such as Tnfα and IL-1β also show a trending increase in expression in the meninges after tMCAO in WT mice. Interestingly, vascular endothelial growth factor (Vegf), a marker of vascular permeability, is also upregulated, indicating a potential role of Mrgprb2 in mediating vascular injury after tMCAO. Put together, deleting Mrgprb2 thus leads to reduced mast cell degranulation and diminished cytokine production, demonstrating that Mrgprb2 acts as a key receptor for meningeal mast cell activation in ischemic stroke.

To further characterize the role of meningeal mast cell activity in stroke, we investigated the meningeal inflammatory environment by analyzing neutrophil populations. We see no differences in meningeal neutrophil counts at an early timepoint of 6 hours, suggesting that early inflammatory injury is no different between WT and *Mrgprb2^−/−^* mice (Figure S2L). However, by 16 hours neutrophil infiltration is elevated in the meninges of WT compared to *Mrgprb2^−/−^* mice (Figure 2G). Curiously, the neutrophil count slowly decreases in WT meninges but rises in *Mrgprb2^−/−^* meninges. By 48 hours, we find significantly fewer neutrophils in the meninges of WT compared to *Mrgprb2^−/−^* mice, and this difference remains beyond 48 hours (Figure S3M–O). This finding indicates that while neutrophils are recruited to the meninges of WT mice early after stroke, they migrate over time and do not accumulate within the meninges. In *Mrgprb2^−/−^* mice, however, neutrophils slowly infiltrate and aggregate over time, indicating that they do not migrate away as dramatically as in WT mice. We thus hypothesized that perhaps meningeal neutrophils in WT mice translocate into the brain, and that this may result in altered immune environments in the ischemic brain. Accordingly, we next studied outcomes of inflammation in WT and *Mrgprb2^−/−^* brains after stroke.

### Mrgprb2^−/−^ mice exhibit attenuated post-stroke inflammation in the brain.

Before assessing inflammation in the brain, we first wanted to assess whether Mrgprb2-mast cells mediate the initial ischemic injury in stroke ^21,23^. We find that there is no significant difference in stroke volume by MRI at 6 hours between WT and *Mrgprb2^−/−^* mice, suggesting that Mrgprb2-mast cells likely do not factor into the acute ischemic injury (Figure S3A). We further demonstrate that both genotypes exhibit similar neuronal injury at 6 hours using glial fibrillary acidic protein (GFAP), an indirect marker of neuronal injury^1,36^, and 4-hydroxynonenal (4-HNE), a marker of oxidative stress after stroke^37,38^ (Figure S3B–C).

**Figure 3.**
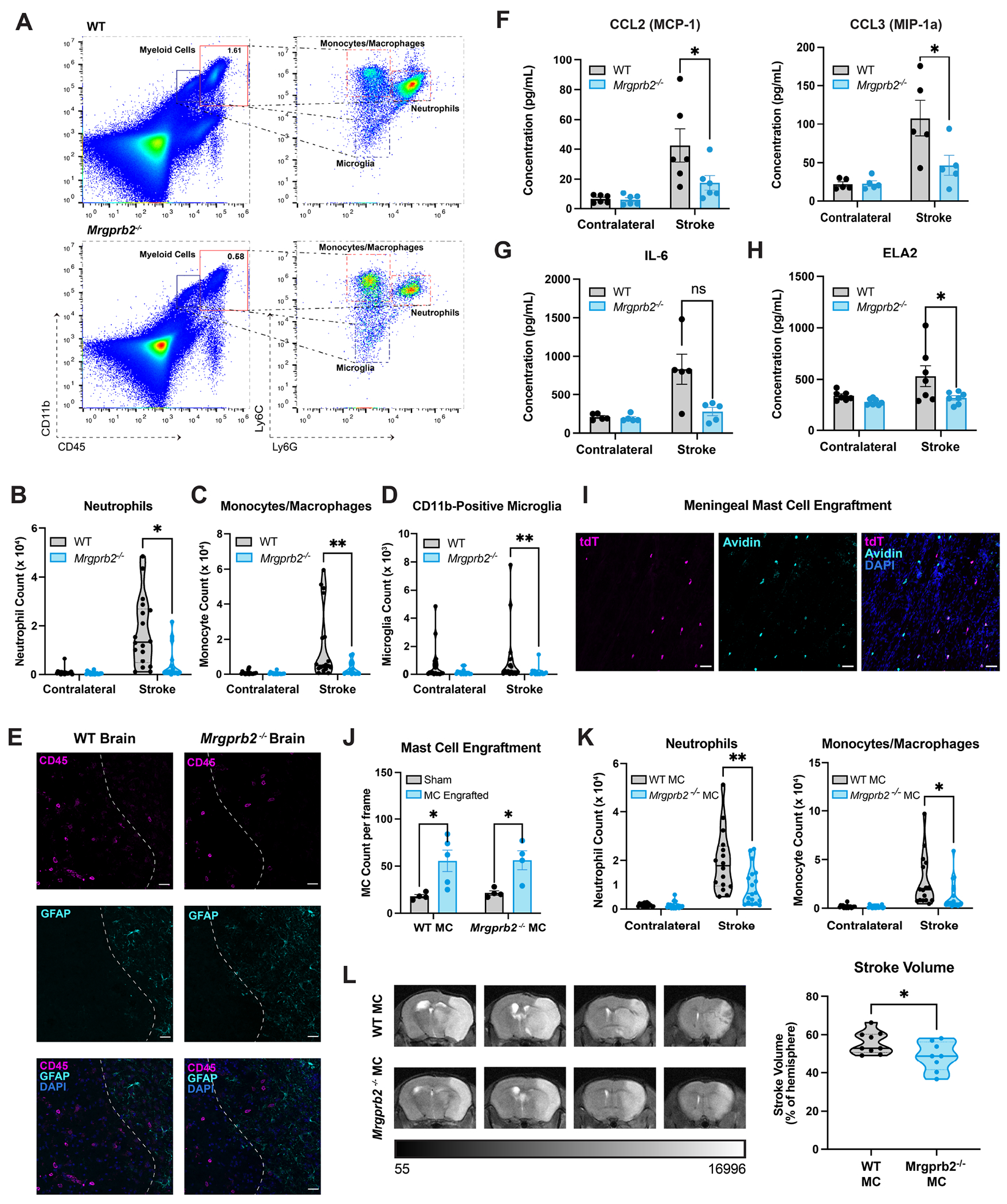
*Mrgprb2*^*−/−*^ mice exhibit reduced brain inflammation after tMCAO, which is rescued by meningeal mast cell engraftment. (A) Representative flow cytometry gating of WT/*Mrgprb2*^*−/−*^ brains 48h post-tMCAO. *left*, Live cells are gated using an immune marker (CD45) and myeloid marker (CD11b). *right*, Myeloid cells are gated using Ly6G and Ly6C to delineate CD45-high neutrophils and monocytes/macrophages, and CD45-low microglia. (B-D) (B) Absolute count of neutrophils, (C) monocytes/macrophages, and (D) CD11b-positive microglia in contralateral/stroke brain hemispheres of WT/*Mrgprb2*^*−/−*^ mice 48h post-tMCAO (WT *n*=18, *Mrgprb2*^*−/−*^ *n*=16). (E) Representative immunofluorescence images of WT (left)/*Mrgprb2*^*−/−*^ (right) right brain hemispheres 48h post-tMCAO. *top row,* CD45-positive immune cells. *middle row,* GFAP-positive activated astrocytes. Dotted white line separates infarcted tissue without GFAP and live, injured brain tissue with GFAP. *bottom row,* Merged channels with DAPI. Scale bar=50 μm. (F), *left,* CCL2 and *right,* CCL3 protein expression measured by ELISA in contralateral/stroke brain hemispheres of WT/*Mrgprb2*^*−/−*^ mice (CCL2: WT *n*=6, *Mrgprb2*^*−/−*^ *n*=6, CCL3: WT *n*=5, *Mrgprb2*^*−/−*^ *n*=5). (G-H) (G) IL-6 and (H) neutrophil elastase protein expression measured by ELISA in contralateral/stroke brain hemispheres of WT/*Mrgprb2*^*−/−*^ mice (G, WT *n*=5, *Mrgprb2*^*−/−*^ *n*=5, H, WT *n*=7, *Mrgprb2*^*−/−*^ *n*=7). (I) Representative immunofluorescence image of *Mrgprb2*^*−/−*^ mouse meninges 8 weeks after engraftment with Mrgprb2-tdT mast cells. *left column,* tdT denotes engrafted cells. *middle column,* Avidin denotes all mast cells. *right column,* Merged channels with DAPI. Scale bar=50 μm. (J) Mast cell count in meninges of *Mrgprb2*^*−/−*^ mice engrafted with saline, WT, or *Mrgprb2*^*−/−*^ mast cells, determined by Avidin-positive cells in the dura, using a 0.408mm^2^ viewing frame (WT saline *n*=4, WT engraftment *n*=5, *Mrgprb2*^*−/−*^ saline *n*=4, *Mrgprb2*^*−/−*^ engraftment *n*=4). (K) Absolute count of neutrophils and monocytes/macrophages in contralateral/stroke brain hemispheres of WT/*Mrgprb2*^*−/−*^ mast cell engrafted mice (WT *n*=16, *Mrgprb2*^*−/−*^ *n*=18). (L). *left,* Representative T2-weighted MRIs, and *right*, stroke volume of WT/*Mrgprb2*^*−/−*^ MC-engrafted *Mrgprb2*^*−/−*^ mice 48h post-tMCAO (WT *n*=9, *Mrgprb2*^*−/−*^ *n*=8). Statistical analyses: Kruskal-Wallis test (B and G), two-way ANOVA with Sidak’s multiple comparisons test (C-D, F, H, and J-K), and two-sided Student’s t-test (L). Statistical test for C, D, and K was performed on log-transformed data to adjust for non-normality. Bar graphs indicate mean ± SEM. *P < 0.05, **P < 0.01, ****P < 0.0001.

To assess the acute immune response after ischemic stroke, we measured immune cell populations in the stroke and contralateral brain hemispheres of WT and *Mrgprb2^−/−^* mice via flow cytometry. We analyzed recruitment of specific innate immune cell populations, first using pan-immune (CD45) and pan-myeloid cell markers (CD11b) and later using cell-specific markers (Ly6G for neutrophils, and Ly6C for monocytes/macrophages) (Figure 3A). We additionally assessed total microglia populations using Iba1 and further identified a subset of CD11b-positive microglia that some have shown to be a reactive population that increases after injury^39^. To further separate microglia from neutrophil and monocyte populations, we ensure that these microglial-subsets are both Ly6G- and Ly6C-negative. No differences in baseline immune cell populations in the brain between WT and *Mrgprb2^−/−^* mice are detected (Figure S3D). Additionally, no differences are noted at an early timepoint of 6 hours after stroke, demonstrating once again that immediate injury is no different between both genotypes of mice (Figure S2E).

We instead show that *Mrgprb2^−/−^* mice exhibit decreased innate inflammation measured by absolute counts of neutrophils, monocytes/macrophages, total microglia, and CD11b-positive microglia in the stroke hemisphere 48 hours after tMCAO (Figure 3B–D, Figure S3F). These differences are recapitulated in WT and *Mrgprb2^−/−^* littermates bred from *Mrgprb2^+/−^ mice* (Figure S3G), which suggests that these distinct immune phenotypes are not artifacts of separate mouse colonies. We demonstrate further by immunofluorescence that these immune cells infiltrate in the stroke hemisphere near the injured, GFAP-positive tissue and do not accrue in the contralateral hemisphere far away from GFAP signal (Figure 3E, Figure S3H).

Commensurate with reduced immune cell recruitment in the brain, we see significantly decreased levels of the chemokines CCL2 and CCL3^40–42^ in *Mrgprb2^−/−^* compared to WT stroke hemispheres (Figure 3F). We additionally show a trending decrease in IL-6 (Figure 3G), a cytokine associated with poor outcomes after ischemic stroke^43–45^, and a significant decrease in neutrophil elastase (NE) (Figure 3H), a neutrophil-specific protease that increases blood brain barrier permeability^46,47^. As seen with brain immune populations, these cytokines and chemokines are only significantly different at 48 hours post-stroke and show no differences in the early injury setting at 6 hours (Figure S3I).

Given that mast cells are known to recruit neutrophils in other inflammatory injuries, we chose to focus on neutrophil regulation in stroke. We previously showed that CXCL2, a master regulator of neutrophil recruitment released by mast cells^48^, was elevated in the meninges of WT mice after tMCAO (Figure 2F). Accordingly, we show that sequestering CXCL2 using a neutralizing antibody injected into the cisterna magna attenuates neutrophil-specific recruitment into the brain after stroke in WT but not *Mrgprb2^−/−^* mice (Figure S3J–K). This decrease is not seen in vehicle-injected mice, suggesting that Mrgprb2 may chemoattract neutrophils via CXCL2. We additionally see that this reduction in neutrophil infiltration into the brain of WT mice is coupled to decreased stroke volume and improved functional outcomes (Figure S3L–M), linking neutrophil-specific recruitment within the brain to worsened stroke outcomes.

Since Mrgprb2 is expressed in connective tissue mast cells beyond the meninges, including the peritoneum, and skin, we wanted to ensure that the effect of Mrgprb2 in mediating brain inflammation is attributed specifically to meningeal mast cells. We thus injected WT or *Mrgprb2^−/−^* cultured mouse mast cells into the meninges of *Mrgprb2^−/−^* mice. First, using Mrgprb2-tdT mast cells injected into non-tdT *Mrgprb2^−/−^* mice, we show that tdT cells are expressed only in the meninges after engraftment (Figure 3I) and that these cells are not in the brain or peritoneum (Figure S3N). We additionally see an increase in meningeal mast cell counts, indicating that the inserted cells have populated within the meninges (Figure 3J). We further confirm by flow cytometry that these cells do not migrate into the brain or peritoneum after tMCAO (Figure S3O). Engrafting *Mrgprb2^−/−^* mice with WT but not *Mrgprb2^−/−^* meningeal mast cells significantly increases brain inflammation after tMCAO as demonstrated by neutrophil and monocyte/macrophage recruitment (Figure 3K). Further, engraftment with WT but not *Mrgprb2^−/−^* meningeal mast cells increases stroke volume in *Mrgprb2^−/−^* mice (Figure 3L). These data suggest that the activity of meningeal-specific Mrgprb2 mast cells is sufficient to incite post-stroke neuroinflammation.

### Mrgprb2 activity regulates immune cell recruitment from the skull bone marrow into the dura.

The skull bone marrow serves as an important reservoir of immune cells for CNS neuroimmunity, providing a source of inflammatory cells specifically for the brain in times of infection and injury^49–51^. New studies have indicated that the skull bone marrow can rapidly expand specifically after ischemic stroke^52^. Additionally, the skull bone marrow and dura are connected by channels which enable the direct transfer of immune cells between these two compartments^53^. Therefore, we hypothesized that Mrgprb2-meningeal mast cells may regulate the recruitment of skull bone marrow immune cells into the dura after stroke.

**Figure 4.**
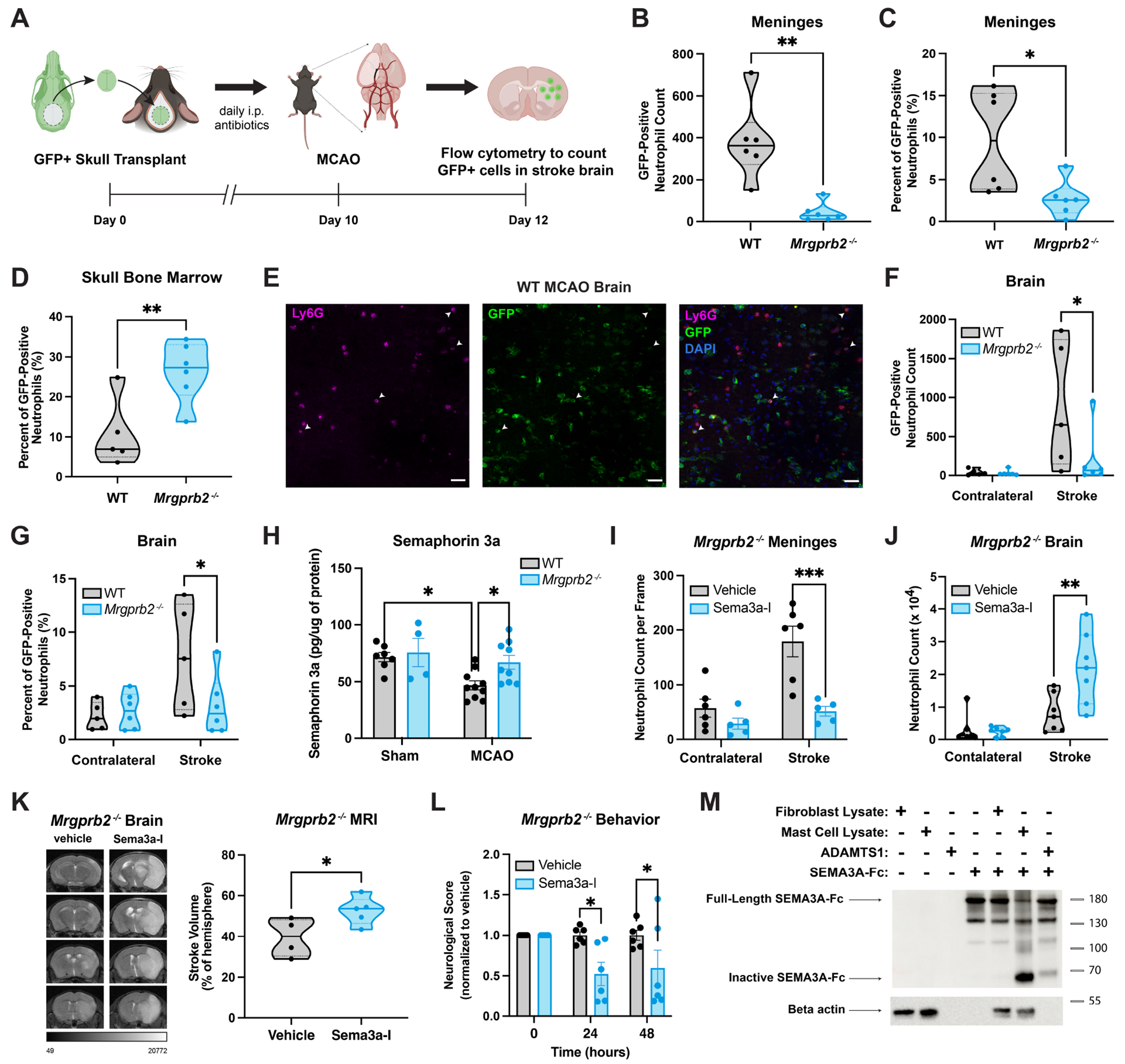
Mrgprb2 is vital for neutrophil recruitment from the skull bone marrow into the brain. (A) Outline of UBC-GFP skull transplant experiment (BioRender). (B-C) (B) Absolute GFP-positive neutrophil count and (C) percentage of dural neutrophils that are GFP-positive in WT/*Mrgprb2*^*−/−*^ mice 48h post-tMCAO (WT *n*=6, *Mrgprb2*^*−/−*^ *n*=6). (D) Percentage of skull bone marrow neutrophils that are GFP-positive in WT/*Mrgprb2*^*−/−*^ mice 48h post-tMCAO (WT *n*=5, *Mrgprb2*^*−/−*^ *n*=6). (E) Representative immunofluorescence image of WT recipient right brain hemisphere 48h post-tMCAO. *left,* Ly6G denotes neutrophils, *middle*, GFP denotes cells recruited from skull bone marrow, and *right,* Merged channels with DAPI. White arrows indicate cells with dual Ly6G/GFP stain. Scale bar=20 μm. (F) Absolute GFP-positive neutrophil count in contralateral/stroke brain hemispheres of WT/*Mrgprb2*^*−/−*^ brains (WT *n*=5, *Mrgprb2*^*−/−*^ *n*=6). (G) Percentage of contralateral/stroke brain hemispheres neutrophils that are GFP-positive in WT/*Mrgprb2*^*−/−*^ brains (WT *n*=5, *Mrgprb2*^*−/−*^ *n*=6). (H) Semaphorin3a protein levels in WT/*Mrgprb2*^*−/−*^ leptomeninges after sham/tMCAO (WT sham *n*=7, *Mrgprb2*^*−/−*^ sham *n=*4, WT tMCAO *n*=10, *Mrgprb2*^*−/−*^ tMCAO *n=*9). (I) Neutrophil count/frame in dura overlaying the contralateral/stroke brain hemisphere of vehicle/Sema3-I treated *Mrgprb2*^*−/−*^ mice 48h post-tMCAO. (vehicle *n*=6, Sema3a-I *n*=5). (J) Absolute neutrophil count in contralateral/stroke brain hemispheres of vehicle/Sema3a-I treated *Mrgprb2*^*−/−*^ mice 48h post-tMCAO (vehicle *n*=7, Sema3a-I *n*=7). (K) *left,* Representative T2-weighted MRIs and *right,* stroke volume of vehicle/Sema3a-I treated *Mrgprb2*^*−/−*^ mice 48h post-tMCAO (vehicle *n*=4, Sema3a-I *n*=5). (L) Vehicle/Sema3a-I treated *Mrgprb2*^*−/−*^ mice neurological scores pre- and post-tMCAO. Scores normalized to vehicle-treated mice within cohorts (vehicle *n*=6, Sema3a-I *n*=6). (M) Recombinant SEMA3A-Fc incubated with dermal fibroblast lysate, WT mast cell lysate, or ADAMTS1 as a positive control. Cleaved, inactive SEMA3A-Fc indicated by smaller 65kDa band. Statistical analyses: Mann-Whitney test (B-C), two-sided Student’s t-test (D, K), two-way ANOVA with Sidak’s multiple comparisons test (F,G,I-J, and L), and two-way ANOVA with Tukey’s multiple comparisons test (H). Statistical test for F, and L was performed on log-transformed data to adjust for non-normality. Bar graphs indicate mean ± SEM. *P < 0.05, **P < 0.01.

We first confirmed that the presence of Mrgprb2 does not alter baseline skull bone marrow immune populations (Figure S4A). To then specifically investigate the role of skull bone marrow recruitment, we transplanted skull flaps from GFP reporter mice (UBC-GFP), onto WT or *Mrgprb2^−/−^* acceptor mice while keeping the dura of the acceptor mice intact (Figure 4A). Since there are no Mrgprb2-mast cells in the skull bone marrow, there is no reintroduction of new populations of mast cells into the acceptor mice (Figure S4B–C). We additionally show that the populations of neutrophils and monocytes within the meninges do not change 10 days after skull transplant, showing that the skull transplant itself does not disrupt the dural immune environment (Figure S4D). GFP signal remains mostly in the skull bone marrow with minimal leakage into the blood, allowing us to selectively track skull bone marrow cells in transplanted mice (Figure S4E). To ensure that the skull transplantation itself did not induce substantial skull bone marrow recruitment, we confirmed that there were no significant differences in meningeal GFP-positive neutrophils and monocytes between genotypes 10 days after transplantation (Figure S4F). We then proceeded with tMCAO in WT and *Mrgprb2^−/−^* acceptor mice and measured GFP signal in the meninges 48 hours later.

We find that the absolute count of GFP-positive neutrophils in the dura of *Mrgprb2^−/−^* mice is significantly reduced after tMCAO (Figure 4B). Specifically, we see that the percentage of total neutrophils in the meninges that are GFP-positive is decreased in *Mrgprb2^−/−^* dura, suggesting that *Mrgprb2^−/−^* mice are unable to recruit the same proportion of skull bone marrow neutrophils into the dura as WT mice (Figure 4C). We similarly see a decrease in the absolute count of GFP-positive monocytes in the dura of *Mrgprb2^−/−^* mice. Although trending, the percentage of recruitment of GFP-positive monocytes into the dura of WT and *Mrgprb2^−/−^* mice is not significantly different, demonstrating that perhaps neutrophils are the main cell recruited at this time (Figure S4G). This GFP-positive neutrophil migration into the dura correlates with a decrease of these cells from the skull bone marrow of WT compared to *Mrgprb2^−/−^* mice (Figure 4D). This change seen in the skull bone marrow is most striking in the neutrophil population, indicating that primarily neutrophils are recruited from this source at this time (Figure S4H). Overall, these data suggest that the presence of Mrgprb2 in the dura may bolster the recruitment of neutrophils from the skull bone marrow reservoir after stroke.

### Mrgprb2 activity mediates migration of dural neutrophils into the brain after stroke.

Previous studies have shown that skull bone marrow-derived immune cells can infiltrate the brain in the acute phase after ischemic stroke^49,50,52^ . We thus wanted to assess whether Mrgprb2 activity can regulate not only skull bone marrow migration into the dura, but also its further infiltration into the brain. We first confirmed that skull transplantation alone without tMCAO does not increase the number of GFP-positive neutrophils or monocytes into the brain parenchyma of donor mice (Figure S4I**).** After tMCAO, we show that many GFP-positive immune cells (Figure S4J) and GFP-positive neutrophils (Figure 4E) infiltrate into the brain 48 hours after stroke. We further demonstrate that WT mice contain significantly higher numbers of GFP-positive neutrophils in the brain parenchyma after tMCAO compared to *Mrgprb2^−/−^* mice (Figure 4F). The ratio of GFP-positive neutrophils to total neutrophils is also significantly higher in WT mice, once again supporting the notion that these mice pull a larger proportion of neutrophils from the skull bone marrow into the brain parenchyma compared to *Mrgprb2^−/−^* mice (Figure 4G). Like the meninges, we see that while the absolute count of GFP-positive monocytes is significantly decreased in *Mrgprb2^−/−^* brains, the percentage of GFP-positive monocytes is not significantly different, suggesting once again that neutrophils may be the primary cell recruited at this time (Figure S4K). We thus establish that Mrgprb2 activity contributes to the migration of neutrophils from the skull bone marrow into the dura, and from the dura into the brain parenchyma after stroke.

### Mrgprb2-mast cell proteases cleave semaphorin, the gatekeeper of dural-brain channels.

While channels that connect the skull bone marrow to the dura have been identified, only recently has it been proposed that bridging veins from the dura to the brain exist that connect through the arachnoid barrier^54^. These veins are surrounded by arachnoid cuff exit points (ACE points), which are gated by semaphorins, a class of chemorepellent molecules that prevent the movement of immune cells through the channels. Decreased expression of semaphorins promotes the migration of immune cells from the dura into the brain. We thus hypothesized that perhaps a reduction of semaphorins after stroke would potentiate movement of dural neutrophils across these bridging points and into the brain. Indeed, we see by ELISA of leptomeningeal lysates that semaphorin 3a (Sema3a), but not semaphorin 3d, is specifically decreased in WT, but not *Mrgprb2^−/−^* mice after stroke (Figure 4H, Figure S4L).

To determine whether this decrease in Sema3a is a driving force for immune cell migration from the dura to the brain, we hypothesized that inhibition of Sema3a should increase neutrophil migration into the brain and exacerbate brain inflammation after stroke. Accordingly, we treated WT and *Mrgprb2^−/−^* mice with a known Sema3a inhibitor (Sema3a-I)^55,56^ in the cisterna magna after tMCAO to inhibit Sema3a at the interface of ACE points. Since the IC_50_ of the Sema3a-I is between 0.1-0.2μM, we treated mice in the cisterna magna with 1μM Sema3a-I at 6 and 24 hours post-tMCAO. As expected, Sema3a inhibition in *Mrgprb2^−/−^* mice reduces neutrophil counts in the dura and increases neutrophil counts in the brain 48 hours after tMCAO (Figure 4I–J). This increase in brain inflammation exacerbates stroke volume (Figure 4K) and worsens functional outcomes in *Mrgprb2^−/−^* mice (Figure 4L), demonstrating that Sema3a inhibition is sufficient to allow *Mrgprb2^−/−^* mice to phenocopy WT mice. Further, Sema3a inhibition does not worsen WT brain inflammation after stroke, suggesting that Sema3a is already inhibited in WT mice after tMCAO (Figure S4M–P). Taken together, these data demonstrate that Sema3a may be inactivated via a Mrgprb2-dependent mechanism.

We further probed how Mrgprb2-mediated mast cell activity may inactivate Sema3a. Previous studies have shown that several proteases, including some expressed by mast cells such as furins and matrix metalloproteinases, can cleave semaphorins^57,58^ into a smaller 65 kilodalton fragment. This cleaved Sema3a is rendered inactive and is unable to function as an immune cell chemorepellent ^59^. We show here that mast cell lysate, but not fibroblast lysate, can significantly cleave full-length Sema3a into its inactive form (Figure 4M), providing a potential mechanism via which Mrgprb2 activation inhibits Sema3a’s chemorepellent activity. To delineate whether the decreased Sema3a seen in WT mice after tMCAO is due to cleavage, we treated leptomeningeal tissue with a known Sema3a protease *ex vivo* and assessed whether cleaved Sema3a can be recognized by ELISA. We show that cleavage of Sema3a reduces its recognition by ELISA (Figure S4Q), indicating that the decrease in Sema3a *in vivo* after tMCAO may be explained by cleavage of Sema3a by mast cell proteases. Collectively, these data support a model in which Mrgprb2-mediated meningeal mast cell activity releases proteases that inactivate Sema3a, disrupting the critical checkpoint and allowing for migration of dural neutrophils through dural-brain channels and into the brain.

### Human meningeal MRGPRX2 mast cells are activated after stroke.

**Figure 5.**
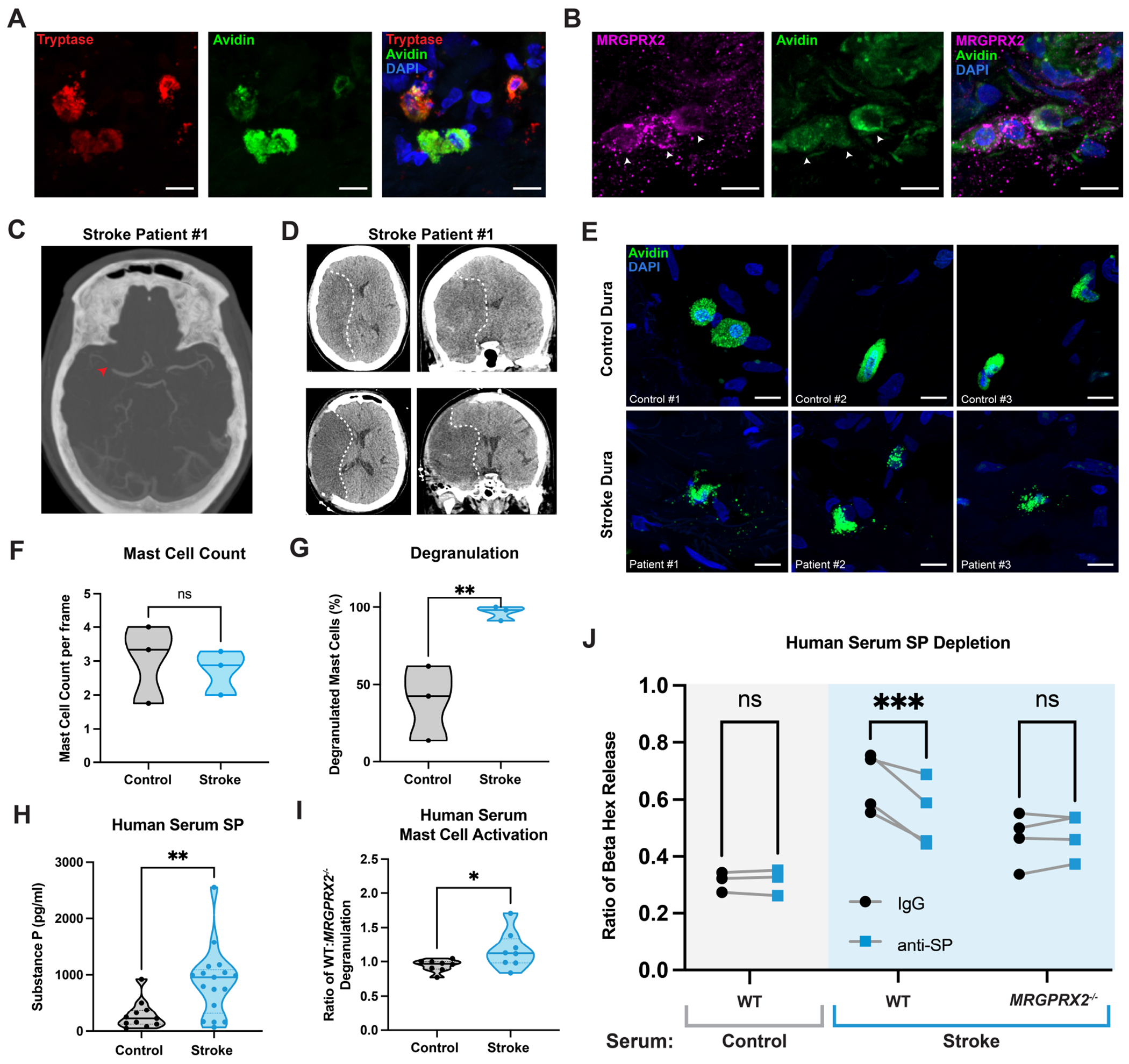
MRGPRX2 mast cells are activated in human stroke dura due in part to substance P. (A) Immunofluorescence images of human control patient dura. *left*, Tryptase and *middle*, Avidin stain mast cells. *right,* Merged channels with DAPI. Scale bar=10 μm. (B) Immunofluorescence image of human dura. *left*, MRGPRX2 stain. *middle*, Avidin co-stain for mast cells. *right,* Merged channels with DAPI. Scale bar=10 μm. (C) CT-angiogram of patient #1 (patient data in Table S1) presenting with right MCA occlusion. Arrow indicates occlusion. (D) *top*, Axial/coronal CT of patient at presentation and *bottom*, after decompressive hemicraniectomy. Dotted lines denote infarcted region. (E) Representative immunofluorescence images of control (*top*) and stroke (*bottom*) patient human dural mast cells stained with Avidin and DAPI. Scale bar=10 μm. Each image denotes distinct patient. (F-G) (F) Mast cells/frame and (G) percentage of degranulated mast cells in control/stroke patient dura. Each point represents one patient (control *n*=3, stroke *n*=3). (H) Substance P protein levels in control/stroke patient serum (control *n*=11, stroke *n*=17). (I) Ratio of WT:*MRGPRX2*^−/−^ LAD2 human mast cell beta-hexosaminidase release using control/stroke human patient serum (control *n*=8, stroke *n*=8). (J) Ratio of WT:*MRGPRX2*^−/−^ LAD2 mast cell beta-hexosaminidase release following incubation with substance P-depleted control/stroke patient serum (control *n*=3, stroke *n*=4). Statistical analyses: two-sided Student’s t-test (**F-G**, and **I**), Mann-Whitney test (**H**), and two-way matched ANOVA with Sidak’s multiple comparisons test (**J**). ns, not significant; *P < 0.05, **P < 0.01, ***P < 0.001.

We next probed whether the human ortholog MRGPRX2 exerts similar pro-inflammatory activity as seen with mouse Mrgprb2. We first collected dura from non-stroke patients undergoing elective craniotomies to determine whether mast cells are present in human dura and whether they express MRGPRX2. We find that avidin and tryptase-expressing mast cells are present in human dura and discover that these cells indeed express MRGPRX2 (Figure 5A–B). We additionally collected post-mortem brain tissue from stroke and non-stroke patients to assess for presence of MRGPRX2-mast cells. While there is an increase in *GFAP* in stroke brains as a marker of injury, there is no *MRGPRX2* or tryptase expression, suggesting a lack of MRGPRX2-expressing mast cells in the human brain after stroke, consistent with our mouse data (Figure S5A, Table S1).

After confirming that these cells are present in human dura, we then sought to determine if these cells play a role in ischemic stroke in human patients. We obtained dura from stroke patients undergoing decompressive hemicraniectomy for malignant middle cerebral artery syndromes (Figure 5C–D, Figure S5B–C, Table S2) and find no differences in the number of mast cells between stroke and control patients. We show, however, that dural mast cells in ischemic stroke patients are significantly more degranulated compared to control patients (Figure 5E–G). This is evidence that human dural mast cells are activated after ischemic stroke in patients.

### Neuropeptide substance P is a ligand for Mrgprb2 and MRGPRX2 activation in stroke.

After determining that Mrgprb2 and MRGPRX2 are key mast cell receptors activated after stroke, we next asked which ligand(s) might underlie its activity. One potential candidate was substance P (SP), a neuropeptide released from neurons upon noxious stimuli and a known agonist of Mrgprb2/X2^21,23^. SP is increased in mouse brains following tMCAO^60–62^, and previous studies have reported that higher serum SP in stroke patients is correlated with higher mortality^63–65^.

We also observe that SP is elevated in the infarcted hemisphere of both WT and *Mrgprb2^−/−^* brains (Figure S5D), indicating that SP is released from injured neuronal tissue after stroke. This localized increase of SP in the stroke hemisphere specifically mirrors the eventual degranulation of meningeal mast cells overlaying the stroke hemisphere seen before in Figure 2E. To determine if SP may drive inflammation via Mrgprb2, SP or vehicle was exogenously injected into the cisterna magna after tMCAO. We see that exogenous SP increases recruitment of neutrophils into the brain of WT but not *Mrgprb2^−/−^* mice, suggesting that SP acts through Mrgprb2 (Figure S5E). Specifically, exogenous SP increases stroke volume in WT mice (Figure S5F). However, this increase in stroke volume is not coupled to worsened behavior in WT mice, reflecting that the endogenous SP released after stroke may be potent enough to elicit strong behavioral outcomes in mice (Figure S5G). However, exogenous SP injection into *Mrgprb2^−/−^* mice neither increases stroke volume nor worsens behavioral outcomes, confirming that SP in stroke acts in an Mrgprb2-dependent manner (Figure S5H–I).

To further assess the role of SP in human stroke, we collected serum samples and observe an increase in serum SP in ischemic stroke patients compared to controls (Figure 5H, Table S3). To determine whether the increase in serum SP activates human mast cells, we exposed cultured human WT and *MRGPRX2^−/−^* LAD2 mast cells to serum from healthy and stroke patients. We find that stroke patient serum activates and degranulates more WT LAD2 mast cells than serum from healthy patients, and that fewer *MRGPRX2^−/−^* mast cells are activated by stroke serum in comparison. *MRGPRX2^−/−^* and WT mast cells show similar reactivity to serum from healthy patients, suggesting that MRGPRX2 is uniquely sensing a factor present in stroke serum (Figure 5I). To narrow down whether MRGPRX2 is responding to SP in the sera, we immuno-depleted both healthy and stroke serum of SP by pre-incubating the sera with anti-SP antibodies (Figure S5J). Clearing SP from stroke serum significantly reduces its activation of WT but not *MRGPRX2^−/−^* LAD2 mast cells, pointing towards a specific SP-MRGPRX2 interaction (Figure 5J). Taken together, we show that human dural mast cells are activated after stroke, and that this activation is in part dependent on SP-mediated activation of MRGPRX2.

### A Mrgprb2 antagonist alleviates post-stroke inflammation and attenuates stroke behavioral deficits and mortality.

**Figure 6.**
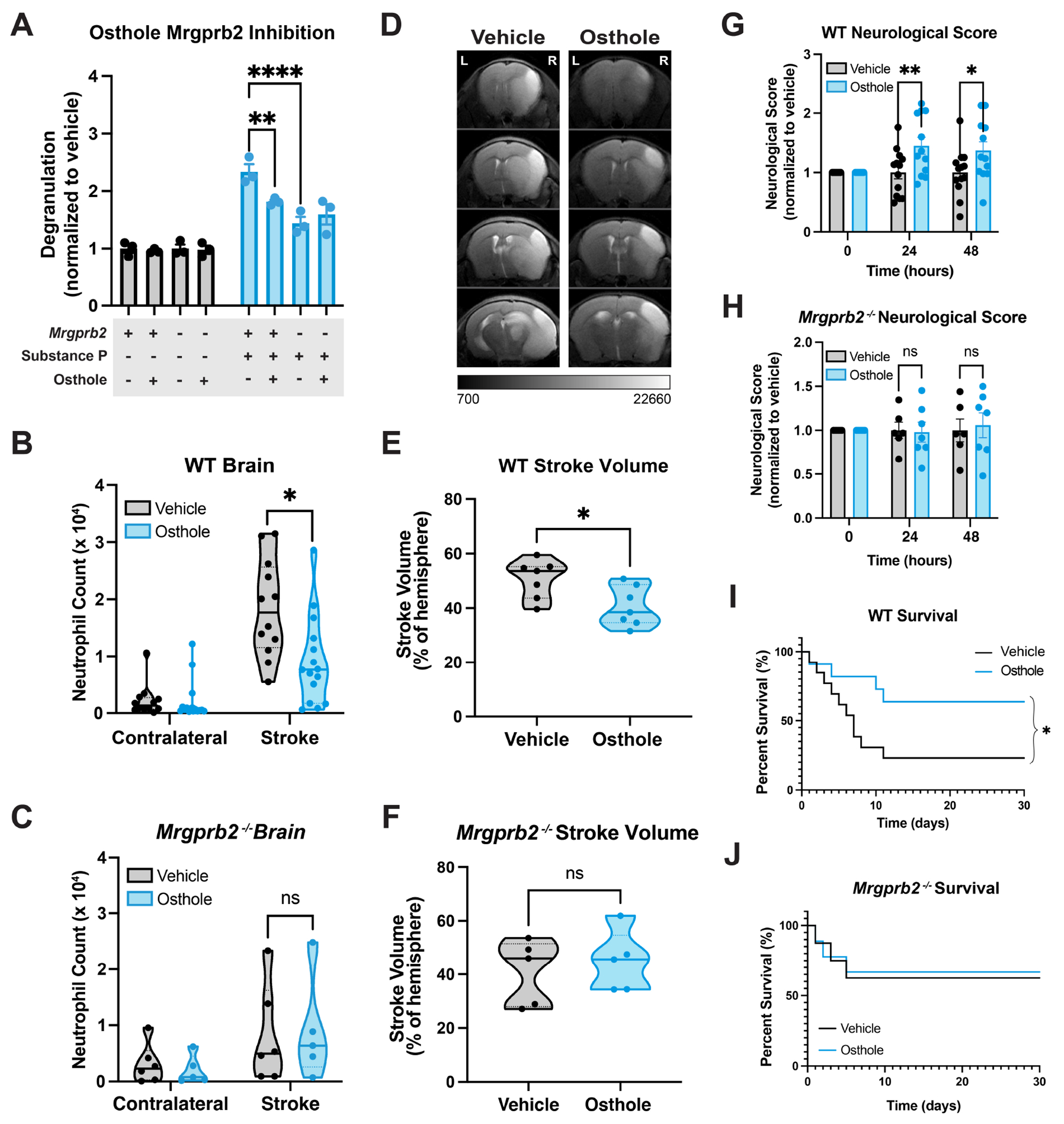
Mrgprb2 inhibition by osthole attenuates post-stroke inflammation and neurologic deficits in mice. (A) Mast cell activity of WT/*Mrgprb2*^*−/−*^ peritoneal mast cells pre-treated with osthole. Data normalized to vehicle of corresponding genotype (*n*=3). (B-C) (B) Absolute neutrophil count in contralateral/stroke brain hemispheres of vehicle/osthole treated WT and (C) *Mrgprb2*^*−/−*^ mice (B, vehicle *n*=12, osthole *n*=15, C, vehicle *n*=5, osthole *n*=7). (D) Representative T2-weighted MRIs of vehicle/osthole treated WT mice. (E-F) Stroke volume in vehicle/osthole treated WT/*Mrgprb2*^*−/−*^ mice 48h post-tMCAO (E, vehicle *n*=7, osthole *n*=7, F, vehicle *n*=5, osthole *n*=5). (G-H) (G) Vehicle/osthole treated WT and (H) *Mrgprb2*^*−/−*^ mice neurological scores pre- and post-tMCAO. Scores normalized to vehicle-treated mice within cohorts (G, vehicle *n*=13, osthole *n*=12, H, vehicle *n*=6, osthole *n*=7). (I-J) Kaplan-Meier survival curves of WT/*Mrgprb2*^*−/−*^ vehicle/osthole treated mice post-tMCAO (I, vehicle *n*=13, osthole *n*=11, J, vehicle *n*=8, osthole *n*=9). Statistical analyses: two-way ANOVA with Tukey’s multiple comparisons test (A), two-way ANOVA with Sidak’s multiple comparisons test (B-C, G-H), two-sided Student’s t-test (E-F), and log-rank test (I-J). Statistical test for B, and C was performed on log-transformed data to adjust for non-normality. Bar graphs indicate mean ± SEM. ns, not significant; *P < 0.05, **P < 0.01, ****P < 0.0001

We next wondered whether pharmacologic inhibition of Mrgprb2 could alleviate post-stroke inflammation and improve stroke outcomes. Osthole, a natural coumarin found in the fruits of *Cnidium monnieri,* is a known Mrgprb2 antagonist^66^. We confirm that pre-treatment of osthole inhibits SP-mediated degranulation in WT but not *Mrgprb2^−/−^* mouse cultured mast cells (Figure 6A). After confirmation of osthole as a Mrgprb2 antagonist, we treated tMCAO mice with two intraperitoneal injections of 100mg/kg osthole^66^ at 6 hours and 24 hours after tMCAO induction. Two days after tMCAO, we assess WT and *Mrgprb2^−/−^* brains for inflammation. We show that osthole-treated WT mice have significantly reduced inflammation by neutrophil count compared to vehicle-treated mice, and that this inflammatory reduction is not seen in *Mrgprb2^−/−^* mice (Figure 6B–C). We additionally determine that monocytes are decreased and CD11b-positive microglia show a trending decrease in the brains of WT but not *Mrgprb2^−/−^* mice after osthole treatment (Figure S6A–B). These data indicate that osthole acts in a Mrgprb2-dependent manner to inhibit post-stroke inflammation. Consistent with previous differences in WT and *Mrgprb2^−/−^* meningeal inflammation at 48 hours after tMCAO, we show that osthole-treated WT mice have more neutrophils in the meninges as these cells cannot traverse into the brain (Figure S6C–D). Osthole-treated *Mrgprb2^−/−^* meninges, however, show no significant differences in neutrophil counts (Figure S6E).

To assess whether this osthole-mediated inhibition of post-stroke inflammation is clinically relevant, we measured stroke infarct volume by MRI. Osthole-treated WT mice show significantly reduced infarct volumes and limited midline shift compared to vehicle-treated mice (Figure 6D–E, Figure S6F). *Mrgprb2^−/−^* mice treated with osthole do not show a further reduction in infarct volume (Figure 6F). Using the 28-point neuroscore, we find that osthole-treated WT but not *Mrgprb2^−/−^* mice exhibit fewer sensorimotor deficits (Figure 6G–H). Lastly, we show that osthole treatment significantly increases survival in WT but not *Mrgprb2^−/−^* mice 30 days after tMCAO, indicating functional efficacy of Mrgprb2 inhibition (Figure 6GI–J). To further confirm that dural Mrgprb2 contributes to post-stroke brain inflammation, we administered a lower dose of osthole locally into the cisterna magna of WT mice at 6 hours and 24 hours after tMCAO. Local osthole injection reduces brain inflammation in WT mice, reduces stroke volume, and improves behavior (Figure S6G–I). Thus, we show here that osthole acts via a Mrgprb2-dependent manner to inhibit post-stroke inflammation and improve outcomes, providing evidence that perhaps inhibition of MRGPRX2 may be a promising therapeutic in combating post-stroke neuroinflammation in human patients.

Together, these data support a model in which Mrgprb2 is a key receptor that allows meningeal mast cells to surveil brain injury in stroke via SP. Once activated, these mast cells act as first responders in initiating recruitment of skull bone marrow immune cells through cytokines such as CXCL2. Mrgprb2 signaling further facilitates the transfer of these immune cells into the brain parenchyma by releasing proteases that inhibit semaphorin activity and promote the migration of immune cells through dural-brain channels. Through these mechanisms, Mrgprb2 signaling promotes mast cell activity in ischemic stroke, contributing towards harmful post-stroke neuroinflammation.

## DISCUSSION

The theory of CNS immune privilege has been slowly replaced by a more complex network of immune homeostasis. The skull bone marrow has been emphasized in recent years as an immune niche that expands over adulthood, ready to activate in times of brain injury^50–53^. Further, the meninges have been supported as an important tissue that directs this complex immune environment, with channels that connect it to the skull bone marrow and to the brain directly^49,54^. Despite this growing evidence of complex immune regulation of the CNS, the signals that orchestrate communication between these tissues remains unknown.

Here, we provide evidence that the mast cell, via its receptor Mrgprb2, is a cell population in the meninges that directs this CNS-immune interaction. While previous studies have established the role of mucosal mast cells^67–69^, a distinct mast population, as well as meningeal mast cells^70–73^, in mediating post-stroke inflammation, none of these data provide a specific mechanism of activation of this cell population, largely limiting clinical translatability. Our finding that Mrgprb2 is a key regulator of these meningeal, connective tissue mast cells alone provides a distinct target to address several diseases and disorders of CNS neuroinflammation.

Beyond this, we introduce a mechanism via which these meningeal mast cells contribute to brain inflammation. Our data suggest that these Mrgprb2-mast cells recruit skull bone marrow neutrophils into the brain, and that the absence of this receptor attenuates this infiltration. Further, by inhibiting the chemorepellent semaphorin 3a, Mrgprb2 disrupts the chemical barrier within dural-brain connections, allowing meningeal neutrophils to migrate into the brain. These data provide a bridge between the skull bone-dura and dura-brain pathways of immune migration, a previously unidentified gap in this important tissue structure.

While semaphorin3a has been reported to function as an inhibitor of immune cell migration^74,75^, exact mechanisms of this activity remain unclear. Previous data supports that semaphorin3a binding to its canonical receptors, neuropilin-1 and plexin a4^76^, can inhibit actin polymerization^77^ , thus preventing motility and migration. Some evidence demonstrates that neutrophils express neuropilin-1^78^ and plexins, providing a possible mechanism via which semaphorin3a can regulate neutrophil migration. Even still, other studies have indicated that semaphorin3a may regulate immune migration through unknown, novel receptors^79^. Thus, further study of the specific receptor via which semaphorin3a modulates neutrophil migration is warranted.

In addition to describing the role of Mrgprb2 in mouse ischemic stroke, we discover that MRGPRX2, the human ortholog of this mast cell receptor, is expressed in human dural mast cells. Moreover, we demonstrate that these human mast cells are activated in response to stroke, and that this activation is in part driven by substance P, a known ligand of MRGPRX2. Therefore, we propose that MRGPRX2 may be a promising drug target in the long search for a therapeutic to combat post-stroke inflammation.

As a promiscuous receptor that can be bound by many positively charged molecules, MRGPRX2 is activated by several pharmaceuticals^21^. Two of these agonists include leuprolide, a gonadotropin-releasing hormone agonist^80,81^, and levofloxacin, an antibiotic in the fluroquinolone class, both of which are known to increase risk of and exacerbate stroke. Specifically, a trial using levofloxacin to limit infections after stroke was prematurely interrupted when it was noted that the levofloxacin treatment arm had worse outcomes than the placebo arm^82^. Exact mechanisms of how these drugs increase and worsen risk of stroke is unclear. Via MRGPRX2, it is plausible that these drugs can activate dural mast cells and thus worsen inflammatory injury in stroke patients. While MRGPRX2 activation may be harmful in stroke patients, inhibition of MRGPRX2 instead may provide robust benefits. Further studies into age- and sex-matched analyses of stroke patients will be imperative to further support this role of MRGPRX2 in stroke.

We have identified MRGPRX2 as a specific target to address post-stroke inflammation, by directly inhibiting the significant mast cell activity that drives the skull bone-dura-brain axis of inflammation. MRGPRX2 expression and activity is confined to the meninges outside of the blood brain barrier, thus allowing ease of access for most drug delivery methods. Thus, MRGPRX2 antagonists are promising potential therapeutic candidates for significantly attenuating post-stroke inflammation. Further studies into the role of this receptor as a regulator of neuroimmunity may additionally uncover a potential role in several diseases and disorders of the central nervous system, centering mast cells as a sentinel immune cell in the meninges that regulates the brain-immune interface.

### LIMITATIONS OF THE STUDY

There are several limitations of this study that warrant further investigation into the role of Mrgprb2 and meningeal mast cells in post-stroke brain inflammation. Firstly, while this study aimed to understand neutrophil recruitment into the brain, there are several immune populations recruited after stroke. Whether this subset of Mrgprb2-expressing meningeal mast cells can influence the recruitment of other cell populations, such as T cells and B cells in chronic inflammatory states after stroke remains to be seen. Secondly, we propose here that the regulation of semaphorin3a by meningeal mast cells may contribute to infiltration of dural neutrophils into the brain. However, whether mast cells regulate other mechanisms of immune cell infiltration into the brain remains to be understood.

## RESOURCE AVAILABILITY

### Lead Contact

Requests for further information and for reagents/resources should be directed to the lead contact, Xinzhong Dong (xdong2@jhmi.edu).

### Materials Availability

Materials generated in this study will be made available upon reasonable request from the lead contact.

### Data and Code Availability

This paper does not report any code or sequencing data. Please refer to supplementary information for the raw western blots. Any other request for original, raw data can be made to the lead contact.

## STAR METHODS

### EXPERIMENTAL MODEL AND STUDY PARTICIPANT DETAILS

#### Mice

All experiments were performed in accordance with protocols approved by the Animal Care and Use Committee at the Johns Hopkins University School of Medicine (Protocol Number: M024M354). All mice were housed in the Miller Research Building animal facility and weaned at 3 weeks of age. Mice were housed with a 12-hour light/dark cycle with controlled temperature and humidity and were fed *ad libitum*. A maximum of 5 mice were housed together in one cage. Mice used for this study were 8–12-week-old males and females on the C57BL/6J background. Prior to tMCAO or sham surgery, mice were otherwise healthy, with no previous procedures or treatments performed. No sex differences were noted, and males and females were used for all experiments. *Mrgprb2*^−/−^ and Mrgprb2-Cre animals were generated as previously described^21^. Littermate controls used in specific littermate experiments as noted above, were generated by breeding *Mrgprb2*^+/−^ mice. UBC-GFP mice used for skull transplantation were purchased from Jackson Laboratories (C57BL/6-Tg(UBC-GFP)30Scha/J, JAX 004353). Surgeons were blinded to mouse genotype during all surgical procedures, and mice were randomized into sham and tMCAO cohorts.

#### Human

##### Institutional Review Board Approval

Patients with ischemic stroke and patients without ischemic stroke undergoing elective craniotomies were recruited under protocols approved by the Institutional Review Board at the Johns Hopkins University School of Medicine Department of Neurosurgery from the dates of November 2022 to January 2024 (study number IRB00336337). Sample sizes for all experiments utilizing human samples can be seen in Tables S1–S3. Both male and female samples were collected. Although the number of human samples was low, we did not notice any sex differences.

##### Dura Collection

Dura from patients undergoing decompressive hemicraniectomy for treatment of ischemic stroke was collected during surgery. Control dura was obtained from elective aneurysm clippings, in which a small piece of dura (5mm by 5mm) is removed. These procedures presented with no clinical symptoms and were therefore used as controls. In both our control and stroke patient procedures, patients are under general anesthesia, positioned supine, and a large craniotomy is performed. Dura is sampled at the beginning of dural opening in both procedures. Table S2 provides the biographical and clinical characteristics of stroke and control cohort dura samples.

Dura was collected in normal saline and placed in 4% paraformaldehyde (PFA) overnight at 4°C. Dura samples were then transferred to 30% sucrose in PBS and incubated overnight at 4°C. They were then transferred to optimal cutting temperature and stored at −80°C until cryosectioned at 10μm sections.

##### Serum

Serum from patients was collected into gold serum separator tubes and centrifuged for 10 min at 1000g within 1 hour of collection. The supernatant was collected and stored at −80°C until used for experiment. Table S3 provides the biographical and clinical characteristics of stroke and control cohorts.

##### Postmortem Brain Tissue

The use of postmortem human samples was deemed exempt by the Committee for the Protection of Human Subjects at the University of Texas Health Science Center at Houston (HSC-MS-22-0982 – UTHealth Neuropathology Core). All participants provided written autopsy consent for the study and subsequent use of the stored samples. Table S1 provides the biographical and clinical characteristics of stroke and control cohorts for postmortem brain tissue samples.

Postmortem human brains were obtained and fixed in 10% neutral buffered formalin for 2–3 weeks, followed by thorough dissection. The dissected brain regions were dehydrated in a graded ethanol series with increasing concentrations, cleared in xylene, and infiltrated with melted paraffin. The paraffin-embedded brain regions were stored at 4°C until sectioning. Tissue sections were cut at a thickness of 5 μm using a rotary microtome, and a total of three sections were pooled for qPCR analysis. RNA was extracted from FFPE sections following manufacturer’s protocol (RNeasy FFPE Kit for RNA extraction, Qiagen). qPCR was then performed as described below.

#### Cells

##### Peritoneal Mast Cell Primary Culture

Adult male and female wild-type and *Mrgprb2^−/−^* mice aged 8 weeks were euthanized through CO_2_ asphyxiation. Peritoneal mast cells (PMCs) were collected after injecting 10 mL of sterile ice-cold phosphate buffered saline (PBS) into the exposed peritoneal cavity. The mouse’s abdomen was massaged gently for 30 seconds. After massaging, the PBS and peritoneal cells were aspirated out of the abdomen and then centrifuged at 300g for 5 min at room temperature. The cell pellet was resuspended in RPMI 1640 Medium (Gibco) with 10% heat-inactivated fetal bovine serum (FBS, Sigma), 100 U/mL penicillin (Gibco), 100 μg/mL streptomycin (Gibco), 30 μg/mL recombinant mouse stem cell factor (Peprotech), and 10 μg/mL recombinant mouse interleukin-3(Peprotech). The cells were transferred into a culture flask to be incubated at 37°C and 5% CO_2_. On every 3^rd^ day for the next 9 days, suspension cells were removed and replaced by fresh medium to promote the proliferation of PMCs. Mast cells were the remaining suspension cells by day 10 and were ready for experiment afterwards. Cells were routinely tested for mast cell identity via flow cytometry.

##### Human LAD2 Mast Cell Culture

LAD2 (Laboratory of Allergic Diseases 2) WT and MRGPRX2^−/−^ male human mast cells were obtained and generated as described previously^86^. Cells were cultured in StemPro-34 SFM medium (Life Technologies) supplemented with 2mM L-glutamine, 100 U/mL penicillin (Gibco), 50μg/mL of streptomycin (Gibco), and 100 ng/mL recombinant human stem cell factor (Peprotech). The cell suspensions were seeded at a density of 1 million cells/mL and maintained at 37°C and 5% CO_2_. Cells are hemi-depleted weekly by removing half of the volume of cells and replenishing with half of fresh media. Cells were routinely tested for mast cell identity via flow cytometry.

### METHOD DETAILS

#### tMCAO Model

Initially, mice were anesthetized using 4% isoflurane and maintained at 1.5% isoflurane during surgery. A rectal probe was inserted to monitor internal body temperature and a heating pad was placed underneath the mouse to maintain the body temperature at 37°C. 70% ethanol was applied around the neck area to disinfect the skin. A midline neck incision was made, and the neck fat pads were pulled apart. The right common carotid artery (CCA) was dissected and an 8-0 silk suture was used to ligate the artery. The external carotid artery (ECA) was dissected free, ligated using two 8-0 silk sutures, and transected. Small surgical scissors were used to cut the ECA in between the 2 ligatures. An aneurysm clip was used to temporarily occlude the distal ICA. A silicone-coated filament (Doccol Corporation) was inserted into the CCA. The filament was advanced into the ICA to obstruct the origin of the middle cerebral artery, as verified by at least 80% decrease in laser doppler intensity. The mice were kept under anesthesia for a 40-minute occlusion time, after which the filament was removed, and the CCA permanently ligated. The skin was sutured using 5-0 sutures and antibacterial ointment (Vetasan Chlorhexide, Valleyvet) was applied. 1mL of normal saline was administered subcutaneously, and 0.5% lidocaine (analgesic) was injected around the incision site. Finally, the mice were placed in a heated cage for 2 hours and given a diet gel for recovery. 1mL of normal saline was administered daily for the following 48 hours. Sham mice underwent the same surgery, however, the filament was inserted and removed immediately before inducing any ischemia. The individual performing tMCAO surgeries was blinded to mouse genotype.

#### Skull Bone Transplant

Skull transplant was carried out as previously described^49^. Mice were anesthetized using 4% isoflurane for induction and 1.5% for maintenance. A rectal probe was inserted to monitor internal body temperature and a heating pad was placed underneath the mouse to maintain body temperature at 37°C. The mouse head was fixed using a stereotactic machine equipped with a mouthpiece that delivers constant oxygen and isoflurane. The mouse head was carefully shaved, then 70% ethanol was applied to disinfect the skin. A midline skin incision was made to expose the skull. Using an electrical microdrill, a piece of the skull centered around the sagittal suture was drilled out. Each flap removed contained portions of the parietal and occipital bones and measured around 4mm in width and 6mm in length. While drilling out the bone the skull was constantly kept wet using a sterile Q-tip and normal saline. The skull flap was then placed in normal saline and incubated at 37°C until the recipient mouse was ready.

WT and *Mrgprb2^−/−^* recipient mice underwent the same procedure as the donor mice. After the recipient skull flap was removed, making sure to keep the underlying dura intact, the donor graft bone was then placed and sealed off carefully using a tissue adhesive glue. The skin was then sutured using 5-0 sutures. 1 mL of normal saline was administered subcutaneously, and 0.5% lidocaine (analgesic) was injected around the incision site. Finally, the mice were placed in a heating cage for 2 hours and given diet gel for recovery. The mice were observed daily and given intraperitoneal injections of Baytril (2.5mg/kg) daily for 10 days.

#### Meningeal Mast Cell Engraftment

Mast cell engraftment into the meninges was performed as previously described^18^. Briefly, mice were anesthetized using 4% isoflurane for induction and 1.5% for maintenance. A rectal probe was inserted to monitor the internal body temperature. A heating pad placed underneath the mice was used to regulate the body temperature, maintained at 37°C. The mouse head was fixed using a stereotactic machine equipped with a mouth piece that delivers constant oxygen and isoflurane. The mouse head was carefully shaved, then 70% ethyl alcohol was applied to disinfect the skin. A midline skin incision was made to expose the skull. Using a microdrill, a small hole that can fit a needle was drilled 1mm lateral to the sagittal suture and 1mm behind the coronal suture on the right parietal bone. A 10 μl Hamilton syringe mounted with a 34G blunt needle was filled with 10μL of 750,000 WT or *Mrgprb2^−/−^* cultured mast cells in sterile saline. The needle was attached to a microinjector set up at a 1μL/min injection rate. Using the stereotactic machine, the Hamilton needle was placed in the drilled hole at a 2mm depth from the skull surface. Once the injection is done the skin was sutured using 5-0 sutures. 1 ml of normal saline was administered subcutaneously. Finally, the mice were placed in a heated cage and given diet gel for recovery.

#### Cisterna Magna Injections

For all cisterna magna (ICM) injections, the protocol was performed as previously described^83^. Briefly, mice were anesthetized using 4% isoflurane for induction and 1.5% for maintenance. The mouse neck was shaved, then wiped with three alternating wipes of betadine and alcohol each. The mouse was placed on an elevated stage, and the head of the mouse was angled downwards at a 45° angle, using the isoflurane nose cone to stabilize the head. The tail of the mouse was taped to the back of the stage. A 32G needle was filled with 10μL of the compound of interest and injected in the gap between the occipital bone and atlas vertebra, until a small pressure was noted, indicating puncture through the atlanto-occipital membrane. The needle was depressed at a rate of 10μL per minute and held within the space for an additional one minute before withdrawal. The mouse was then placed in a heated cage and monitored for signs of distress. The following doses of each substance were given at 6 hours and 24 hours post-tMCAO: vehicle (10μL of varied vehicles), substance P (10μL of 1 mM stock in saline), anti-CXCL2 antibody (10μL of 0.5 mg/mL in saline), anti-rat IgG antibody (vehicle control for CXCL2 experiments: 10μL of 0.5 mg/mL in saline), Sema3a-I (10μL of 1uM stock in 0.1% DMSO in saline), and osthole (1mg/kg in 10uL of 1% DMSO,1% Tween-80 in saline).

#### Behavioral Tests

##### Rotarod:

For the rotarod test, each mouse was trained for 5 minutes at a constant speed of 4 rpm. This training period was repeated three times per day for three consecutive days before experiment. On experimental days, each mouse underwent three trials. Each trial started at 4 rpm and constantly accelerated to 40 rpm by 5 minutes. The trial ended when the mouse fell off and this time was recorded as the latency to fall (in seconds).

##### Catwalk:

The Catwalk system (Noldus) was used for gait analysis. Mice were trained on the Catwalk for two consecutive days before experiment. Training consisted of allowing the mouse to roam freely in the walkway for 5 minutes each day. On experimental days, each mouse was allowed to roam freely in the walkway, and recording was ended when the mouse traversed the length of the walkway three times. After recording, the toe spread of each paw for each mouse was measured using the Noldus software.

##### 28-Point Neuroscore:

Mouse behavioral sensorimotor function was quantified using the 28-point Neuroscore test as described previously^84^. Subjects were acclimated to the testing environment, and behavioral deficits were assessed at baseline, 24- and 48-hours post-MCAO. Scoring was averaged between two independent scorers who were blinded to mouse genotype. 11 total tests were performed on each day, with a maximum score of 28 points indicating no behavioral deficits. The lowest score for each test was 0 points, with the maximum score ranging between 1-4 depending on the test. For each test, higher scores indicated better performance. Mice that received scores below 7 (indicative of minimal movement and activity) were excluded from experiment.

The tests were performed as described below:

1) Mouse behavior was observed throughout the test cage and assessed on whether it displayed circling behavior. 2) Motility of each mouse was assessed, with increasing degrees of unsteadiness corresponding to a lower score. 3) The general condition of each mouse was inspected for scoring: poor grooming, hunched posture, and weak muscle tone indicated worse condition and corresponded to lower scores. 4) The mouse was lifted by the tail and held so that its body would be parallel to the edge of a table. Successful paw placement on the left and right side were assessed. 5) To test for the righting reflex, the mouse was manually positioned in the cage in a supine position on the floor and assessed for its ability to orient upright. 6) The mouse was placed with its forepaws on the wire bar cage top so that it would be allowed to hang. The mouse’s ability to grab onto the cage and raise its hindlimbs was assessed. 7) Grip strength was observed and scored based on the previous set-up in test 6. 8) The mouse was positioned to face downward on top of the test cage lid, which was placed at a 45° angle. The mouse lost points if it was unable to rotate to face upward quickly (within 20 seconds). 9) The mouse was held by the tail and assessed for the curling reflex, with curling indicating behavioral deficit. 10) The mouse was positioned so its head would be just below the edge of the table. Mice received lower scores if they were unable to arch their back and reach to place both forepaws on the tabletop. 11) The mouse was held by the tail and rotated clockwise and counterclockwise. Mice were given points if they were able to swivel contralaterally to the rotation direction. All behavioral tests were performed by the same individual who was blinded to genotype.

#### MRI

Mice were anesthetized with 2.5% isoflurane and continually placed at 2% isoflurane for the duration of imaging. Brain MR imaging was performed on a 7T/30 MRI scanner (Bruker BioSpin, Billerica, MA, USA) using a 72mm Tx volume array and a mouse brain Rx surface coil. Axial T2-weighted TubroRARE MRI sequences covering the whole brain were taken using the following parameters: TE/TR of 27ms/3000 ms, resolution of 0.1 × 0.1 mm, 15 sections, FOV: 20X20mm; 1mm section thickness, matrix size of 200 × 200, and four averages with rare factor 8 were performed in all mice. The total MR scan time was around 7 minutes per mouse.

#### TTC

For chemical stain of stroke infarct, 2% 2,3,5-triphenyltetrazolium (TTC) was dissolved in PBS (2g/100mL). Mice were perfused and whole brains were extracted and cut into 6 sections with a 2mm thickness. Olfactory bulb and cerebellum were removed from staining. Each 2mm brain section was placed in 1mL of 2% TTC and allowed to stain for 5 minutes protected from light. Each section was then washed with 1mL of PBS and placed on a flat glass surface. The anterior and posterior sides of each section were imaged.

#### Stroke Infarct Quantification

For both TTC and MRI images, infarct size was quantified as previously described^85^. Briefly, for each section, three areas were measured using ImageJ: infarct area, ipsilateral hemisphere area (infarcted + non-infarcted region), and contralateral hemisphere area. Each area was then multiplied by the thickness of the section. For MRI, the section thickness was 1mm. For TTC, the anterior and posterior sides of each section were measured separately and thus the section thickness was also 1mm. To determine an edema-adjusted stroke infarct volume, the following formula was used:

$$Stroke\;Volume\left(\%\right)=100*\frac{contralateral-\left(ipsilateral-stroke\right)}{contralateral}$$

#### Midline Shift Quantification

To measure the midline shift, the T2 MRI coronal view of a section where the third ventricle is clearly visible was chosen. Using ImageJ, the width of the brain section was measured using a horizontal line from one edge of the brain to the other, passing through the third ventricle. The width of the stroke hemisphere was also measured using a horizontal line at the same dorsal/ventral position as the original line. The following formula was then used to calculate percent midline shift:

$$Shift\;from\;Midline\left(\%\right)=-1*100*\frac{stroke\;hemisphere\;width}{brain\;width}$$

#### Osthole Preparation and Administration

A stock solution of osthole (TCI America) was prepared at 125mg/mL in DMSO. For each mouse, a 100mg/kg dose of osthole was given intraperitoneally with the following formulation: 10% DMSO, 10% Tween-80, in normal saline. Mice were injected 6 hours after tMCAO, and 24 hours after tMCAO.

#### Beta hexosaminidase degranulation assay

##### Human LAD2 Cells

50μL of 5 x 10^5^ cells/mL of WT and MRGPRX2^−/−^ cells were seeded per well in a 96-well round bottom plate. 50μL of 2X treatment was then added to the cells, to obtain a final 1X concentration in the well. Substance P was treated using a 100nM final concentration. All serum samples were first cleared by centrifugation and added directly to each well, for a final 1:2 dilution in the well. Cells were treated for 30 minutes then spun down at 300g for 5 min. 50μL of supernatant was removed and added to a 96-well assay plate. 50μL of cell lysis buffer (0.1% Triton-X in PBS) was then added to the remaining cell and supernatant wells and mixed thoroughly. Once mixed, 50μL of this solution was added to the other half of the 96-well assay plate. 50μL of p-nitrophenyl N-acetyl-β-D-glucosaminide (Sigma-Aldrich) in 0.1 M sodium citrate buffer (pH 4.5) was added to each well for 90 minutes and incubated at 37°C. After incubation, the reaction was stopped by adding 50μL of 0.4M pH 10.7 glycine buffer to each well. The plate was immediately read at absorbance 405nm with 570nm as reference. The percent of beta hexosaminidase released was calculated by the following formula:

$$Beta\;Hex\;Release\left(\%\right)=100*\frac{\text{supernatant}\;\text{absorbance}}{0.5\;*\;\text{supernatant}\;\text{absorbance}\;+\;\text{cell}\;\text{absorbance}}$$

##### Mouse Peritoneal Mast Cells

50μL of 2 x 10^6^ cells/mL of WT and *Mrgprb2^−/−^* cells were seeded per well in a 96-well round bottom plate. For pretreatment with osthole, cells were treated with 25μL of 3X osthole (final concentration in well: 100μM in 0.01% DMSO) for 1 hour at 37°C. Cells were then treated with 25μL of 4X substance P (final concentration of 50μM) for 30 minutes at 37°C. Beta hexosaminidase protocol was followed exactly as above after treatment.

#### Substance P Depletion

To deplete human serum of substance P, anti-rat IgG2a (ThermoFisher Scientific) and rat anti-SP (Millipore Sigma) antibodies were first separately incubated with Protein A/G beads (50μg of antibody in 100μL of Protein A/G slurry) for 2 hours at room temperature on a shaker. Excess antibody was then washed away from the beads with 3, 5-minute washes in 1mL of PBS on the shaker, followed by centrifugation at 3000g for 3 minutes. 50μL of human serum was then incubated with 10μL of IgG-conjugated beads or anti-SP-conjugated beads overnight at 4°C on a shaker. The following day, beads were spun down at 3000g for 3 minutes and the supernatant was separated from the bead pellet. This supernatant was then used for degranulation assays with LAD2 mast cells as described above.

#### Semaphorin 3a Cleavage Assay

WT LAD2 mast cells and human dermal fibroblasts were lysed with 0.1% Triton-X (without protease inhibitors). Protein was estimated and normalized between both samples. 40μL of each sample was added to 0.75μg of recombinant human semaphorin 3A-Fc in 1.5mL tubes (Sino Biological). A negative control of semaphorin on its own was placed in 40μL of 0.1% Triton-X. For positive control, 0.75μg of recombinant ADAMTS1 (R&D Systems) was placed with 0.75μg of semaphorin in 40μL of 50mM Tris pH 7.4, 10mM CaCl_2_, 80mM NaCl. All reactions were placed at 37°C overnight. After incubation, 10μL of SDS-loading buffer was added to each sample and boiled at 100°C for 5 minutes before loading for western blot analysis.

#### Flow Cytometry

##### Brain

Adult mice were anesthetized with 200μL of 20% urethane in saline and perfused with 20 mL of phosphate buffered saline (PBS) (pH 7.4, 4°C). After perfusion, the brain was dissected, and the right and left hemispheres were divided. Tissues were dounce homogenized in 1 mL of brain digestion buffer (HBSS containing 10% fetal bovine serum, 5mg/mL Collagenase IV, and 30μg/mL DNase I) and brought up to a volume of 4mL with digestion buffer. Samples were digested for 20 min at 37°C in a rotisserie incubator. Following incubation, samples were passed through a 70 μM mesh cell strainer and 5 mL of PBS was added to the suspension and centrifuged at 450g for 5 min at 4°C. The supernatant was discarded, and the pellet was resuspended in 30% Percoll (Sigma-Aldrich) in PBS and spun down at 900g for 35 min at 4°C. The upper layer was gently aspirated and the pellet was given a final wash in 5 mL of PBS and spun down at 450g for 5 min at 4°C before moving to flow staining.

##### Dura

The dura was peeled from the skull and processed by mincing in 1mL of digestion buffer (DMEM containing 2% fetal bovine serum, 1mg/mL Collagenase VIII, and 30μg/mL DNase I). They were then incubated for 20 min at 37°C in a rotisserie incubator and passed through a 70 μM cell strainer. The samples were spun down (300g, 5 min, 4°C) and washed in 1mL of PBS before flow staining.

##### Skull Bone Marrow

The skull was minced into small pieces, dounce homogenized with a pestle in 1mL of PBS, and filtered through a 70 μM cell strainer. The cells were centrifuged at 300g for 5 min at 4°C and 1mL of ACK was added to each sample to dissolve red blood cells. After incubation for 5 min at room temperature, the cells were centrifuged (300g, 5 min, 4°C) and resuspended in 5mL of PBS. Cells were centrifuged and resuspended in FACs buffer for flow staining.

##### Peritoneal Fluid

The peritoneal fluid was processed by injecting 10 mL of PBS into the exposed peritoneal cavity and aspirating the fluid. The peritoneal fluid was spun down at 300g for 5 min at 4°C and washed in 5mL of PBS before moved to flow staining.

Once digested, each sample was resuspended in 1mL of Live/Dead Fixable Aqua Dead Cell Stain Kit (1:1000, Invitrogen). Cells were centrifuged (300g, 5 min, 4°C) and resuspended in Fc Block (1:100; BioLegend) in FACs buffer and incubated at room temperature for 5 minutes. Cells were then stained with the following antibodies for 25 minutes at 4°C protected from light: anti-CD45 APC-Cy7 (1:200, BioLegend), anti-CD117 Brilliant Violet 605 (1:50, BioLegend), anti-FcεRIα Pe/Cy7 (1:50, BioLegend), anti-CD11b Pe/Dazzle594 (1:200, BioLegend), anti-Ly6G Brilliant Violent 421 (1:100, BioLegend), anti-Ly6C APC (1:100, BioLegend). After staining, cells were washed with 1mL of FACs buffer and resuspended in 300μL FACs buffer (containing 50μL of CountBright Absolute Counting beads) before running on the BD FACSCelesta Flow Cytometer. For samples treated with intracellular antibodies, cells were first stained with cell-surface antibodies as mentioned above, then washed. Cells were then fixed for 20 minutes at room temperature with Cyto-Fast Fix/Perm Solution (Biolegend), followed by two washes in 1X Cyto-Fast Wash buffer. Cells were stained with anti-Iba1 (1:100, Abcam) for 10 minutes at room temperature, followed by secondary stain in goat anti-mouse IgG AF488 (1:500, Invitrogen) for 10 minutes at room temperature. Cells were then washed once with 1X Cyto-Fast Wash buffer and resuspended in 300μL FACs buffer (containing 50μL of CountBright Absolute Counting beads) before running on the BD FACSCelesta Flow Cytometer. Cell counts were normalized using the absolute bead count collected on the machine and analyzed using FlowJo (TreeStar).

#### Protein Quantification by ELISA

Brain tissue was dissected after perfusion, and a midsagittal transection was performed to separate the stroke and contralateral hemispheres. The samples were snap-frozen in liquid nitrogen and homogenized in 500μL of RIPA lysis buffer (50 mM Tris-HCl, 150 mM NaCl, 0.5% Sodium deoxycholate, and 1% Triton X-100) containing a 1:100 concentration of protease inhibitors (Sigma-Aldrich). The samples were centrifuged at maximum speed for 15 minutes at 4°C, and supernatants were harvested. Total protein concentration was measured using the Pierce BCA Protein Assay (ThermoFisher Scientific). 50μg of lysate was used for each ELISA analysis. Substance P, IL-6, ELA2, CCL2 (MCP-1), and CCL3 (MIP-1a) levels were measured using ELISA DuoSets (R&D Systems) according to the manufacturer’s instruction. For human serum substance P quantification, human serum was diluted 1:2 and assayed using the SP Parameter Assay ELISA Kit (R&D Systems).

#### Semaphorin ELISA

Leptomeningeal semaphorin ELISAs were performed as previously described^54^. Briefly, the mouse brain was dissected and placed in a dish containing ice-cold PBS. The leptomeninges were then extracted from the dorsal hemispheres of the brain using Dumont #5 forceps. Once extracted, the leptomeninges were placed in 50μL of PBS containing protease inhibitors (1:100), then freeze-thawed three times in liquid nitrogen. The suspension was then centrifuged at 14,000g for 10 minutes, and the supernatant was collected for assay. Protein concentration was determined by BCA assay and 10μg of each sample was loaded into the ELISA plate. The ELISA was carried out by manufacturer’s instructions.

Sema3a ELISA on cleaved Sema3a in leptomeningeal lysates were tested as followed. After leptomeninges were extracted, they were placed directly in 50μL of PBS with or without 2μg of a Sema3a protease, ADAMTS1. Leptomeninges were then incubated overnight at 37°C to allow for protease activity. Supernatants were then separated from leptomeningeal tissue, and the leptomeninges were then lysed using three freeze-thaw cycles in liquid nitrogen. Lysates were then centrifuged at 14,000g for 10 minutes, and the supernatants were collected for assay. Sema3a detected in supernatants of overnight cleavage were normalized to Sema3a detected in the corresponding cell lysates.

#### Western Blot

For western blot analysis, tissues were dounce homogenized with RIPA lysis buffer (50 mM Tris-HCl, 150 mM NaCl, 0.5% Sodium deoxycholate, and 1% Triton X-100) containing a 1:100 concentration of protease inhibitors (Sigma-Aldrich). Lysates were then allowed to shake at 4°C for 20 min, then pulse-sonicated and centrifuged at 16,000g for 15 min at 4°C. Protein concentration was measured using the BCA assay (ThermoFisher Scientific). Western samples were made using 15 μg of lysate, 6.25 μl 4x LDS sample buffer (Invitrogen), 1 μl Dithiothreitol (DTT), and Phosphate-buffered saline (PBS) to a total volume of 25uL. Samples were then heated at 100°C for 5 min, then centrifuged briefly. Western samples were run on a 4-12% polyacrylamide Bis-Tris gradient gel in 1x running buffer (Invitrogen) and then transferred to a PVDF membrane. Membranes were blocked with 5% milk in TBS-T (pH 7.6 solution of 16 mM Tris-HCl, 140 mM NaCl, 0.1% Tween-20) and incubated with primary antibodies in 3% bovine serum albumin (BSA) in TBST overnight at 4°C. Following incubation in primary antibody, membranes were washed with TBS-T, then incubated with secondary antibodies (1:10,000) in 3% BSA in TBS-T for 2 h at room temperature. The following primary antibodies were used: anti-GFAP (1:1,000; Abcam), anti-ACTB HRP conjugated (1:10,000; Santa Cruz Biotechnology), and anti-4HNE (1:1,000; Abcam).

#### RNA isolation and quantification

Meningeal tissue was carefully harvested from WT and *Mrgprb2^−/−^* adult mice. Total RNA for the meningeal samples was extracted using the RNeasy^®^ Plus Micro Kit (Qiagen), according to the manufacturer’s instructions. Tissue lysate was sonicated with 3, 10-second pulses right after addition of RNA extraction buffer. RNA was quantified using NanoDrop. Reverse transcription was performed using the iScript cDNA synthesis kit and following the manufacturer’s suggestions. Finally, qPCR was performed using TaqMan^™^ Fast Advanced Master Mix (Applied Biosystems) and run in triplicate on a StepOnePlus Real-Time PCR System (Applied Biosciences) and analyzed by StepOne Software v2.2.2. Gene expression was normalized using a Mrgprb2 probe that recognizes both WT and mutant Mrgprb2 and calculated with 2-Δnormalized ^Ct^. Actin could not be used as a loading control since there are differences in total cell count due to inflammation, however since mast cell counts do not change, Mrgprb2 was used as a normalization control. It is important to note that the Mrgprb2 mRNA probe binds in a region distant from the Mrgprb2 mutation that causes lack of translation of Mrgprb2 in the *Mrgprb2^−/−^* mice. Thus, this probe can determine general transcription of Mrgprb2, even though it is not translated in the *Mrgprb2^−/−^* mice.

#### Immunofluorescence

##### Mouse Brain

Adult mice were anesthetized with 200μL of 20% urethane in saline and perfused with 20 mL of phosphate buffered saline (PBS), followed by 20mL of 4% paraformaldehyde (PFA). After perfusion, the brain was dissected and post-fixed in 4% paraformaldehyde (PFA) at 4°C overnight, followed by overnight incubation in 30% sucrose at 4°C. After removal of sucrose the brain was then stored in optimal cutting temperature compound (OCT) at −80°C until sectioned with a cryostat at 20uM sections onto slides. Sections were washed with PBS to remove any remaining OCT, then placed in blocking solution (10% normal goat serum in PBS, 0.2% Triton-X) for 1 h at room temperature. Sections were then incubated in antibody overnight in blocking solution at 4°C as follows: anti-GFAP (1:1000; Abcam), anti-CD45 (1:100; BioLegend), anti-Ly6G (1:200; BioLegend), anti-GFP (1:100; Aves Labs). Sections were then washed with PBS (3 washes, 5 min each) and incubated in secondary antibody in blocking solution at room temperature for 2h. All secondary antibodies (Life Technologies) were diluted 1:500, except for anti-chicken 488 which was diluted at 1:1000. Slides were then washed with PBS (5 washes, 5 min each), mounted with Fluoromount-G with DAPI (Invitrogen), and imaged using the Zeiss LSM700 Confocal microscope.

##### Mouse Whole Mount Dura

Adult mice were anesthetized with 200μL of 20% urethane in saline and perfused with 20 mL of phosphate buffered saline (PBS) (pH 7.4, 4°C). After perfusion, the skull cap with dura mater is dissected and post-fixed in 4% paraformaldehyde (PFA) at 4°C. After 1 hour, the dura was then carefully peeled back from the skull cap in a PBS solution and transferred gently to a slide, ready for staining. Whole mounts were washed with PBS, then permeabilized with 0.5% Triton-X in PBS for 45 min at room temperature. Tissue was then blocked in blocking solution (10% normal goat serum in PBS, 0.2% Triton-X) for 1 h at room temperature, before primary antibody incubation in blocking solution overnight at 4°C as follows: anti-Ly6G (BioLegend; 1:200), anti-CD31 (1:100; BD Pharmingen). Tissues are then washed with PBS (3 washes, 5 min each) and incubated in secondary antibody in blocking solution at room temperature for 2 h. All secondary antibodies are diluted 1:500. Avidin stain is also performed during secondary antibody incubation in blocking buffer for 2 h at room temperature (Avidin-FITC, Invitrogen/Avidin-Sulforhodamine, Abcam ; 1:500). Slides were then washed with PBS (5 washes, 5 min each), mounted with Fluoromount-G with DAPI (Invitrogen), and imaged using the Zeiss LSM700 Confocal microscope.

##### Human Dura

Human dura was obtained and placed in 4% PFA overnight at 4°C. Dura was then transferred to 30% sucrose for overnight incubation at 4°C. After removal from sucrose, dura was stored in optimal cutting temperature compound (OCT) at −80°C until sectioned with a cryostat at 10μM sections onto slides. Slides were washed with PBS to remove excess OCT and then placed in blocking solution (10% normal goat serum, 0.3% Triton-X) for 1h at room temperature. If slides were also receiving Avidin stain, they were first placed in Avidin (1:500) in blocking solution for 2h at room temperature. Slides were then washed with PBS (3 washes, 5 min each), before incubation in primary antibody in blocking solution overnight at 4°C as follows: anti-MRGPRX2 (1:200; BioLegend), anti-tryptase (1:500; Santa Cruz Biotechnology). Slides were then washed with PBS (3 washes, 5 min each), before incubation in secondary antibody solution as described above for 2h at room temperature. Slides were then washed (5 washed, 5 min each), mounted with Fluoromount-G with DAPI (Invitrogen), and imaged using the Zeiss LSM700 Confocal microscope. MRGPRX2 antibody validated on human WT and MRGPRX2^−/−^ cultured mast cells.

#### Mouse Meningeal Neutrophil Quantification

Neutrophil count in the meninges is calculated by dividing the meninges from anterior to posterior into three equally portioned sections, excluding the cerebellar meninges. Within each hemisphere (right and left), one image is taken per section, and the neutrophils in each image are manually counted. The representative neutrophil count per hemisphere is determined by summing the three sections. These counts were done by two individuals blinded to genotype and counts are reported as an average of the two measurements.

#### Mouse/Human Meningeal Mast Cell Degranulation Quantification

Several confocal images (between 3-7) are taken from each hemisphere of the whole mount dura sample per mouse. The total number of mast cells across all images is counted and the number of mast cells that are degranulated across all images is counted. Degranulated mast cells are identified as cells that have at least 5 extracellular vesicles, defined as small circles between 0.8-1.5μm in size (to ensure they are not background signal/autofluorescence), within a 5μm distance of the cell membrane. These numbers are then summed across all images per hemisphere of one sample and the percent of total mast cells that are degranulated is determined. These counts were done by two individuals blinded to genotype and counts are reported as an average of the two measurements. The same protocol is used for mast cell degranulation quantification in human tissue, except that there is no hemisphere delineation.

### QUANTIFICATION AND STATISTICAL ANALYSIS

Statistical analyses were performed using GraphPad Prism v.10.0.2. Statistical comparisons were conducted by two-tailed, unpaired Student’s t-test, or two-way ANOVA with Sidak’s multiple comparison test or Tukey’s multiple comparison test, unless otherwise noted. All data were tested for normality where applicable. If data did not follow a normal distribution, the appropriate nonparametric test was performed. Accordingly, the Mann-Whitney test was used in place of the unpaired student’s t-test. In non-normally distributed cases where two-way ANOVA was the appropriate statistical test, data were first transformed using a log-scale and then reanalyzed for normality and two-way ANOVA testing. The Kruskal-Wallis test was further used if transformed data did not follow a normal distribution. For all bar charts, bars depict the mean. For all violin plots, bold lines depict the mean. All error bars represent SEM and n represents the number of mice analyzed. Specific details of each experiment, including statistical test used and exact n value, can be found in the figure legends.

## Supplementary Material

Supplementary Data S1 Data S1. Raw western blot images of GFAP and 4-HNE, related to Figure S3.

Supplementary Data S2 Data S2. Raw western blot images of semaphorin cleavage, related to Figure 4.

Supplementary Figure S1 Figure S1 related to Figure 1. *Mrgprb2^−/−^* mice exhibit equivalent blood flow reduction during tMCAO but have improved neurological outcomes.
(A) Blood flow reduction during 40-minute occlusion time measured by laser doppler. Baseline blood flow measured before surgery is 100% (WT *n*=23, *Mrgprb2^−/−^ n*=22).
(B) Brain midline shift determined by MR imaging 48h after stroke. Negative shift indicates midline of the brain shifted away from the right (stroke) hemisphere into the left hemisphere. Whiskers indicate minimum and maximum values, and bold line depicts the mean (WT *n*=8, *Mrgprb2*^−/−^ *n*=11).
(C) *left*, Right front paw and *right,* right hind paw toe spread measured by Catwalk for WT/*Mrgprb2^−/−^* mice pre- and post-tMCAO (WT *n*=4, *Mrgprb2*^−/−^ *n*=4).
Statistical analyses: two-sided Student’s t-test (A), Mann-Whitney test (B), and two-way ANOVA with Sidak’s multiple comparisons test (C). Bar graphs indicate mean ± SEM. ns, not significant; **P < 0.01.

Supplementary Figure S2 Figure S2 related to Figure 2. Mrgprb2 shows 100% penetrance in mast cells of the meninges and *Mrgprb2^−/−^* mice show significant differences in neutrophil accumulation within the meninges after stroke.
(A) Whole mount dura from Mrgprb2-Cre;tdT mice. *left,* Avidin signal identifies mast cells. *middle,* tdT signal correlates to Mrgprb2 expression. *right,* Avidin and tdT co-localize in every mast cell. DAPI is used to identify nuclei. Scale bar=500 μm.
(B) Zoom of whole mount dura. Same colors as in A. Scale bar=25 μm.
(C-E) (C) Peritoneal fluid, (D) dural meninges, and (E) whole brain from Cre− and Cre+ mice. Cre− mice do not express tdT and are used as negative control to properly gate each tissue for positive tdT signal. *left*, All CD45-positive immune cells from each tissue are plotted. Gray histogram is Cre− tissue, red histogram is Cre+ tissue. Number indicates frequency of Cre+ immune cells that are tdT positive. *right*, tdT positive cells gated for mast cells using CD117 and FcER1α. Number is frequency of tdT cells that are mast cells.
(F) Flow cytometry of Mrgprb2-Cre;tdT peritoneal fluid, and whole brain 48h post-tMCAO. Each whole brain panel denotes a distinct mouse. Live cells gated for CD45 and tdT. Number in each quadrant indicates frequency of that population in all live cells.
(G) Representative sections from anterior to posterior of one Mrgprb2-Cre;tdT mouse brain 48h after stroke. *top*, tdT fluorescence indicates Mrgprb2-expressing cells. No tdT positive cells are seen. *bottom,* GFAP indicates activated astrocytes that surround infarcted brain tissue. Scale bar=1000 μm.
(H) Mast cell count in WT/*Mrgprb2^−/−^* mice in sham and tMCAO mice 48h after surgery, determined by avidin positive cells in the dura. Count is per frame using a 2.24mm^2^ viewing frame (WT sham *n*=3, *Mrgprb2^−/−^* sham *n*=3, WT tMCAO *n*=3, *Mrgprb2^−/−^* tMCAO *n*=5).
(I) Average expression of mRNA using single cell analysis of the whole brain using the Brain Cell Data Viewer (https://www.braincelldata.org/singlecell)^33^. No Mrgprb2 expression is seen in any cell of the brain. (J) Percent of degranulated mast cells in WT/*Mrgprb2^−/−^* sham meninges in dura overlaying the contralateral and ipsilateral brain hemispheres (WT *n*=4, *Mrgprb2*^−/−^ *n*=4).
(K) Relative mRNA expression of various genes in WT/*Mrgprb2^−/−^* dura 48h post-tMCAO, normalized to WT sham gene expression (WT sham *n*=4, *Mrgprb2^−/−^* sham *n*=4, WT tMCAO *n*=3, *Mrgprb2^−/−^* tMCAO *n*=4).
(L) Neutrophil count per frame in WT/*Mrgprb2^−/−^* dura overlaying the contralateral and stroke brain hemisphere 6h post-tMCAO (WT *n*=6, *Mrgprb2^−/−^ n*=6).
(M) Neutrophil count per frame in WT/*Mrgprb2^−/−^* dura overlaying the contralateral and stroke brain hemisphere 72h post-tMCAO. (WT *n*=6, *Mrgprb2^−/−^ n*=6).
(N) Neutrophil counts in WT/*Mrgprb2^−/−^* dura overlaying the stroke hemisphere at different timepoints from 6 to 72h post-tMCAO. These data are replotted from Fig. 2G and Figure S2L–M to show a clearer time-course of neutrophil recruitment.
(O) Representative immunofluorescence images of WT/*Mrgprb2^−/−^* right hemispheric dura 6, 16, 24, 48, and 72h post-tMCAO. Ly6G denotes neutrophils and DAPI identifies nuclei. Scale bar=50 μm.
Statistical analyses: two-way ANOVA with Sidak’s multiple comparisons test (H, J-N). Statistical test for K (IL-1β expression), L, and N was performed on log-transformed data to adjust for non-normality. Bar graphs indicate mean ± SEM. ns, not significant; *P < 0.05, **P < 0.01, ***P < 0.001.

Supplementary Figure S3 Figure S3 related to Figure 3. WT/*Mrgprb2^−/−^* mice demonstrate similar ischemic injury immediately after stroke but show significant differences in neutrophil accumulation after stroke.
(A) Quantification of stroke volume in WT/*Mrgprb2^−/−^* mice 6h post-tMCAO (WT *n*=6, *Mrgprb2*^−/−^ *n*=5).
(B-C) Western blot and quantification of (B) GFAP (main band at 49 kDa) and (C) 4-hydroxynonenal in WT/*Mrgprb2^−/−^* brains 6h post-tMCAO (WT *n*=3, *Mrgprb2*^−/−^ *n*=3).
(D) Flow cytometry of baseline WT/*Mrgprb2^−/−^* whole brains without tMCAO surgery. Cell counts of neutrophils, monocytes/macrophages, CD11b-positive microglia, and total microglia are denoted (WT *n*=3, *Mrgprb2*^−/−^ *n*=3).
(E) Absolute count of *left,* neutrophils, *middle,* monocytes/macrophages, and *right,* CD11b-positive microglia in contralateral and stroke brain hemispheres of WT/*Mrgprb2^−/−^* mice 6h post-tMCAO (WT *n*=6, *Mrgprb2*^−/−^ *n*=7).
(F) Absolute count of total microglia in contralateral and stroke brain hemispheres of WT/*Mrgprb2^−/−^* mice 48h post-tMCAO (WT *n*=6, *Mrgprb2*^−/−^ *n*=6).
(G) Absolute count of *left,* neutrophils, *middle,* monocytes/macrophages, and *right,* CD11b-positive microglia in contralateral and stroke brain hemispheres of WT/*Mrgprb2^−/−^* littermate mice (WT *n*=8, *Mrgprb2*^−/−^ *n*=5).
(H) Representative immunofluorescence images of *left,* WT, and *right, Mrgprb2^−/−^* contralateral brain hemispheres 48h post-tMCAO. CD45 denotes immune cells, GFAP counterstain delineates activated astrocytes, and DAPI identify nuclei. Scale bar=50 μm.
(I) Protein expression measured by ELISA of various cytokines and chemokines in contralateral and stroke brain hemispheres of WT/*Mrgprb2^−/−^* mice 6h post-tMCAO (CCL2: WT *n*=6, *Mrgprb2^−/−^ n*=6, CCL3: WT *n*=6, *Mrgprb2^−/−^ n*=6, IL-6: WT *n*=4, *Mrgprb2^−/−^ n*=5, ELA2: WT *n*=6, *Mrgprb2^−/−^ n*=6).
(J) Absolute count of neutrophils in the brain at 48h in *left,* WT and *right, Mrgprb2^−/−^* mice injected with vehicle or anti-CXCL2 antibody at 6h and 24h post-MCAO in the cisterna magna (WT vehicle *n*=5, WT anti-CXCL2 *n*=6, *Mrgprb2^−/−^* vehicle *n*=3, *Mrgprb2^−/−^* anti-CXCL2 *n*=6).
(K) Absolute count of monocytes/macrophages at 48h in *left,* WT and *right, Mrgprb2^−/−^* mice injected with vehicle or anti-CXCL2 antibody at 6h and 24h post-MCAO in the cisterna magna (WT vehicle *n*=5, WT anti-CXCL2 *n*=6, *Mrgprb2^−/−^* vehicle *n*=3, *Mrgprb2^−/−^* anti-CXCL2 *n*=6).
(L) Quantification of stroke volume in vehicle and anti-CXCL2 antibody treated WT mice 48h post-tMCAO (vehicle *n*=7, anti-CXCL2 *n*=7).
(M) Vehicle and anti-CXCL2 antibody treated WT mice neurological scores pre- and post-tMCAO. Scores normalized to vehicle-treated mice within cohorts (vehicle *n*=7, anti-CXCL2 *n*=9).
(N) *left,* Peritoneal fluid, and *right,* brain of *Mrgprb2^−/−^* mice 8 weeks after engraftment with Mrgprb2-Cre tdT mast cells. Gray histogram indicates cells with no tdT expression (same gating as in Extended Data Figure 2). Horizontal bar gates cells that are positive for tdT expression.
(O). Percentage of immune cells in each tissue compartment of Mrgprb*2^−/−^* mice engrafted with Mrgprb2-Cre tdT mast cells (gray box). Red bar indicates positive control of percentage of immune cells in Mrgprb2-Cre+ tdT peritoneum without engraftment that express tdT. All other bars refer to tissues in the engrafted mouse, 48h post-tMCAO (tdT Control *n=*3, Mrgprb*2^−/−^* engrafted mice *n*=4).
Statistical analyses: two-sided Student’s t-test (A-B, L), Mann-Whitney test (C), two-way ANOVA with Sidak’s multiple comparisons test (D-G, H-K, M), Kruskal-Wallis test (I, ELA2, J, Mrgprb*2^−/−^*) and ordinary one-way ANOVA (O). Statistical test for E (neutrophils), and K (Mrgprb*2^−/−^*) was performed on log-transformed data to adjust for non-normality. Bar graphs indicate mean ± SEM. ns, not significant; *P < 0.05, **P < 0.01, ***P < 0.001, ****P < 0.0001.

Supplementary Figure S4 Figure S4 related to Figure 4. GFP-positive cells predominate in the skull bone marrow after skull transplantation.
(A) Percent of *left,* neutrophils and *right,* monocytes among all immune cells in the skull bone marrow in WT/*Mrgprb2^−/−^* mice (WT *n*=7, *Mrgprb2^−/−^ n=*8).
(B) Percent of Mrgprb2-tdT mast cells in all immune cells among peritoneal fluid, meninges, and skull bone marrow (SBM) (n=3 mice per tissue).
(C) Mast cell count in WT/*Mrgprb2^−/−^* mice at baseline and 10 days after skull transplant determined by avidin positive cells in the dura. Count is per frame using a 0.408mm^2^ viewing frame (WT baseline/transplant *n*=3, *Mrgprb2^−/−^* baseline/transplant *n*=3).
(D) Percent of *left*, neutrophils and *right*, monocytes among all immune cells in the meninges of WT/*Mrgprb2^−/−^* mice at baseline at 10 days after skull transplant (WT baseline *n=*4, *Mrgprb2^−/−^* baseline *n*=4, WT transplant *n*=4, *Mrgprb2^−/−^* transplant *n*=4).
(E) *left,* Percent of GFP-positive immune cells, *middle*, GFP-positive neutrophils, and *right,* GFP-positive monocytes in the blood and SBM 10 days after skull transplant in WT/*Mrgprb2^−/−^* mice (WT *n=*6, *Mrgprb2^−/−^ n*=7).
(F) Percentage of *left,* GFP-positive neutrophils and *right,* GFP-positive monocytes in WT/*Mrgprb2^−/−^* meninges after skull transplant and 48h after sham surgery (WT *n*=6, *Mrgprb2^−/−^ n*=7).
(G). *left,* Absolute count of GFP-positive monocytes and *right*, percentage of total monocytes that are GFP-positive in dura of WT/*Mrgprb2^−/−^* mice 48h post-tMCAO (WT *n*=6, *Mrgprb2^−/−^ n*=6).
(H) Percentage of *left,* total immune cells and *right,* total monocytes that are GFP-positive in skull bone marrow of WT/*Mrgprb2^−/−^* mice 48h post-tMCAO (WT *n*=5, *Mrgprb2^−/−^ n*=6).
(I) Absolute count of GFP-positive *left,* neutrophils and *right,* monocytes in contralateral and ipsilateral brain hemispheres after skull transplant and 48h after sham surgery (WT *n*=6, *Mrgprb2^−/−^ n*=7).
(J) Representative immunofluorescence images of WT recipient right brain hemisphere 48h post-tMCAO. *top,* GFP denotes cells recruited from skull bone marrow. Arrows indicate GFP-positive cells. *middle,* CD45 denotes all immune cells. Arrows point to all GFP-positive cells from previous image. *bottom,* CD45 and GFP co-stain, along with DAPI to identify nuclei, indicates that all GFP-positive cells co-stain with CD45, indicating they are immune cells. Scale bar=25 μm.
(K) *left,* Absolute count of GFP-positive monocytes and *right*, percentage of total monocytes that are GFP-positive in contralateral and stroke brain hemispheres of WT/*Mrgprb2^−/−^* brains 48h post-tMCAO (WT *n*=6, *Mrgprb2^−/−^ n*=7).
(L) Protein levels of semaphorin 3d in WT/*Mrgprb2^−/−^* sham and tMCAO leptomeninges 48h post-tMCAO measured by ELISA (WT sham *n*=3, *Mrgprb2^−/−^* sham *n*=3, WT tMCAO *n*=9, *Mrgprb2^−/−^* tMCAO *n*=8).
(M) Neutrophil count per frame in dura overlaying the contralateral and stroke brain hemisphere of vehicle and Sema3a-I treated WT mice 48h post-tMCAO. (vehicle *n*=4, Sema3a-I *n*=5).
(N) Absolute count of neutrophils in contralateral and stroke brain hemispheres of vehicle and Sema3a-I treated WT mice 48h post-tMCAO (vehicle *n*=8, Sema3a-I *n*=10).
(O) Quantification of stroke volume in vehicle and Sema3a-I treated WT mice 48h post-tMCAO (vehicle *n*=5, Sema3a-I *n*=4).
(P) Vehicle and Sema3a-I treated WT mice neurological scores pre- and post-tMCAO. Scores normalized to vehicle-treated mice within cohorts (vehicle *n*=5, Sema3a-I *n*=5).
(Q) Protein levels of semaphorin3a in leptomeninges treated with protease inhibitors (control) or known Sema3a protease ADAMTS1. Protein amount in supernatant normalized to protein amount in lysate (control *n*=4, ADAMTS1 *n*=5).
Statistical analyses: two-sided Student’s t-test (A, G-H, and Q), two-way ANOVA with Sidak’s multiple comparisons test (C-D, I, K, M-N, and P), Kruskal-Wallis test (E), Mann-Whitney test (F, O), and two-way ANOVA with Tukey’s multiple comparisons test (L). Statistical test for K (absolute count), M, and P was performed on log-transformed data to adjust for non-normality. Bar graphs indicate mean ± SEM. ns, not significant; *P < 0.05, **P < 0.01, ***P < 0.001, ****P < 0.0001.

Supplementary Figure S5 Figure S5 related to Figure 5. Human stroke brain tissue does not express *MRGPRX2*, and Substance P is increased in the stroke mouse brain and human stroke serum.
(A) Relative mRNA expression of *MRPRX2* (left), tryptase (middle), and *GFAP* (right), in control and stroke patient brain tissue, normalized to β-actin expression. LAD2 WT and LAD2 KO cells serve as positive and negative controls for *MRGPRX2* expression, and as positive controls for *TPSB2* expression (*MRGPRX2*: control *n*=8, stroke *n*=24, *TPSB2*: control *n*=10, stroke *n=2*6, *GFAP* control *n*=7, stroke *n*=20).
(B) *left,* CT-angiogram of patient #2 (patient data in Table S2) presenting with left ICA termination occlusion. Arrow pointing at site of occlusion. *right*, *top,* Axial and coronal CT images of patient at time of presentation and *bottom*, axial and coronal CT images of patient after decompressive hemicraniectomy. Dotted lines denote infarcted region.
(C) *left,* CT-angiogram of patient #3 (patient data in Table S2) presenting with left supraclinoid ICA occlusion. Arrow pointing at site of occlusion. *right*, *top*, Axial and coronal CT images of patient at time of presentation and *bottom*, axial and coronal CT images of patient after decompressive hemicraniectomy. Dotted lines denote infarcted region.
(D) Quantification of substance P neuropeptide measured by ELISA in WT/*Mrgprb2^−/−^* contralateral and stroke brain hemispheres 48h post-tMCAO (WT *n*=6, *Mrgprb2^−/−^ n*=6).
(E) Absolute count of neutrophils at 48h in *left,* WT and *right, Mrgprb2^−/−^* injected with vehicle or SP at 6h and 24h post-MCAO in the cisterna magna (WT vehicle *n*=6, WT SP *n*=6, *Mrgprb2^−/−^* vehicle *n*=6, *Mrgprb2^−/−^* SP *n*=6).
(F) Quantification of stroke volume in vehicle and SP treated WT mice 48h post-tMCAO (vehicle *n*=8, SP *n*=10).
(G) Vehicle and SP treated WT mice neurological scores pre- and post-tMCAO. Scores normalized to vehicle-treated mice within cohorts (vehicle *n*=9, SP *n*=9).
(H) Quantification of stroke volume in vehicle and SP treated *Mrgprb2^−/−^* mice 48h post-tMCAO (vehicle *n*=4, SP *n*=5).
(I) Vehicle and SP treated *Mrgprb2^−/−^* mice neurological scores pre- and post-tMCAO. Scores normalized to vehicle-treated mice within cohorts (vehicle *n*=4, SP *n*=5).
(J) Substance P measured in serum of control and stroke patients after incubation with anti-IgG or anti-SP antibodies (control *n*=6, stroke *n*=6).
Statistical analyses: two-way ANOVA with Sidak’s multiple comparisons test (A, E, G, and I), two-way ANOVA with Tukey’s multiple comparisons test (D), Mann-Whitney test (F), two-sided Student’s t-test (H), and two-way matched ANOVA with Sidak’s multiple comparisons test (J). Statistical test for G, and I was performed on log-transformed data to adjust for non-normality. Bar graphs indicate mean ± SEM. ns, not significant; *P < 0.05, **P < 0.01, ***P < 0.001, ****P < 0.0001.

Supplementary Figure S6 Figure S6 related to Figure 6. Osthole reduces inflammation and injury in WT but not *Mrgprb2^−/−^* mice.
(A) Absolute count of *left,* monocytes/macrophages and *right*, CD11b-positive microglia in contralateral and stroke brain hemispheres of WT vehicle and osthole treated mice 48h tMCAO (vehicle *n*=12, osthole *n*=15). (B) Absolute count of *left,* monocytes/macrophages and *right*, CD11b-positive microglia in contralateral and stroke brain hemispheres of *Mrgprb2^−/−^* vehicle and osthole treated mice 48h post-tMCAO (vehicle *n*=5, osthole *n*=7).
(C) Representative immunofluorescence images of WT *left,* vehicle and *right,* osthole treated right hemispheric dura 48h post-tMCAO. Ly6G denotes neutrophils and DAPI identifies nuclei. Scale bar=50 μm. (D-E) Neutrophils in vehicle and osthole treated (D) WT and (E) *Mrgprb2^−/−^* dura 48h post-tMCAO (WT vehicle *n*=4, WT osthole *n*=6, *Mrgprb2^−/−^* vehicle *n*=6, *Mrgprb2^−/−^* osthole *n*=6).
(F) Brain midline shift determined by MR imaging 48h after stroke. Negative shift indicates midline of the brain shifted away from the right (stroke) hemisphere into the left hemisphere. Whiskers indicate minimum and maximum values, and bold line depicts the mean (vehicle *n*=7, osthole *n*=7).
(G) Absolute count of *left,* neutrophils, *middle,* monocytes/macrophages, and *right,* activated CD11b-positive microglia in contralateral and stroke brain hemispheres of cisterna magna vehicle and osthole treated WT mice 48h post-tMCAO (vehicle *n*=8, osthole *n*=11).
(H) Quantification of stroke volume in cisterna magna vehicle and osthole treated WT mice 48h post-tMCAO (vehicle *n*=6, osthole *n*=6).
(I) Cisterna magna vehicle and osthole treated WT mice neurological scores pre- and post-tMCAO. Scores normalized to vehicle-treated mice within cohorts (vehicle *n*=10, osthole *n*=10).
Statistical analyses: two-way ANOVA with Sidak’s multiple comparisons test (A-B, D-E, G, and I) and two-sided Student’s t-test (F and H). Statistical test for A, B, D, and G (monocytes) was performed on log-transformed data to adjust for non-normality. Bar graphs indicate mean ± SEM. ns, not significant. * *P* < 0.05, ** *P* < 0.01.

Supplementary Tables S1-S3 Table S1 Related to Figure 5: Postmortem brain patient characteristics used in qPCR analysis.
Table S2 Related to Figure 5: Demographic and clinical presentation of ischemic stroke patients and controls used in human dura analyses.
**Table S3 related to** Figure 5**: Demographic and clinical presentation of ischemic stroke patients and controls used in human blood analyses**. Data are presented as number of patients (% of total).

## Figures and Tables

**Table:** Key resources table

REAGENT or RESOURCE	SOURCE	IDENTIFIER
Antibodies		
APC anti-mouse Ly-6C	BioLegend	Cat #: 128016; RRID: AB_1732076
APC/Cyanine7 anti-mouse CD45	BioLegend	Cat #: 103116; RRID: AB_312981
Brilliant Violet 421 anti-mouse Ly-6G	BioLegend	Cat #: 127628; RRID: AB_2562567
Brilliant Violet 605 anti-mouse CD117	BioLegend	Cat #: 135122; RRID: AB_2562042
PE/Dazzle 594 anti-mouse/human CD11b	BioLegend	Cat #: 101256; RRID: AB_2563648
PE/Cyanine7 anti-mouse FcεRIα Antibody	BioLegend	Cat #: 134318; RRID: AB_10640122
Purified anti-mouse Ly-6G	BioLegend	Cat#: 127602; RRID: AB_1089180
PE anti-mouse CD45	BioLegend	Cat#: 103106; RRID: AB_312971
Alexa Fluor 700 anti-mouse CD206	BioLegend	Cat#: 141734; RRID: AB_2629637
PE anti-mouse F4/80	BioLegend	Cat#: 123110; RRID: AB_893486
FITC anti-mouse/human CD11b	BioLegend	Cat#: 101206; RRID: AB_312789
Rabbit anti-Human IgG Fc, HRP	Invitrogen	Cat#: 31423; RRID: AB_228409
Rabbit polyclonal anti-Semaphorin 3A	Abcam	Cat#: ab23393; RRID: AB_447408
Chicken polyclonal anti-GFAP	Abcam	Cat#: ab4674; RRID: AB_304558
β-Actin HRP	Santa Cruz Biotechnology	Cat #: sc-47778; RRID: AB_626632
Mouse monoclonal anti-mast cell tryptase	Santa Cruz Biotechnology	Cat #: sc-59587; RRID: AB_793510
Purified rat anti-mouse CD31	BD Biosciences	Cat#: 550274; RRID: AB_393571
Avidin, Fluorescein Conjugate	Sigma-Aldrich	Cat#: 189727; RRID: N/A
Avidin sulforhodamine 101 conjugate	Abcam	Cat#: ab275308; RRID: N/A
Purified anti-human MRGPRX2	BioLegend	Cat#: 359002; RRID: AB_2562299
Rat IgG2a Isotype Control	Thermo Fisher	Cat#: 02-9688; RRID: AB_2532970
Anti-substance P	Millipore Sigma	Cat#: MAB356; RRID: AB_94639
Anti-4 Hydroxynonenal	Abcam	Cat#: ab46545; RRID: AB_722490
Anti-Iba1	Abcam	Cat#: ab283319; RRID: AB_2924797
Alexa Fluor 488 goat anti-rabbit IgG	Thermo Fisher	Cat#: A-11008; RRID: AB_143165
Alexa Fluor 488 goat anti-rat IgG	Thermo Fisher	Cat#: A-11006; RRID: AB_2534074
Alexa Fluor 594 goat anti-rabbit IgG	Thermo Fisher	Cat#: A-11012; RRID: AB_2534079
Alexa Fluor 594 goat anti-mouse IgG	Thermo Fisher	Cat#: A-11005; RRID: AB_2534073
Anti-mouse IgG HRP	Cytiva	Cat#: NA931; RRID: AB_772210
Anti-rabbit IgG HRP	Cytiva	Cat#: NA934; RRID: AB_772206
CXCL2 Monoclonal Antibody	Thermo Fisher	Cat#: MA5-23737; RRID: AB_2609513
Rat IgG2a Isotype Control	Thermo Fisher	Cat#: 02-9688; RRID: AB_2532970
Biological samples		
Patient dura and blood samples: control and stroke	Johns Hopkins School of Medicine	N/A
Human brain samples: control and stroke	UT Health Houston	N/A
Chemicals, peptides, and recombinant proteins		
Semaphorin-3A/SEMA3A Protein, Mouse	MedChemExpress	Cat#: HY-P74566
Recombinant Human Semaphorin 3A Protein (hFc Tag)	SinoBiological	Cat#: 10758-H01H-20
Recombinant Human ADAMTS1 Protein	R&D Systems	Cat#: 2197-AD
Collagenase IV	Worthington Biochemical Corporation	Cat#: 9001-12-1
Collagenase VIII	Millipore Sigma	Cat#: C2139
Deoxyribonuclease I	Worthington Biochemical Corporation	Cat#: 9003-98-9
Pierce Protein A/G Magnetic Beads	Thermo Scientific	Cat#: 88803
Osthole	TCI Chemicals	Cat#: 00426
Substance P	Tocris	Cat#: 1156
Vinaxanthone (Sema3a Inhibitor)	MedChemExpress	Cat#: HY-N9480
Recombinant Murine SCF	PeproTech	Cat#: 250-03
Recombinant Murine IL-3	PeproTech	Cat#: 213-13
Critical commercial assays		
Substance P Parameter Assay Kit	R&D Systems	Cat#: KGE007
Mouse Semaphorin 3A ELISA Kit	Cusabio	Cat#: CSB-EL020980MO
Mouse Semaphorin 3D ELISA Kit	Cusabio	Cat#: CSB-EL020983MO
Mouse CCL2 ELISA	R&D Systems	Cat#: DY479
Mouse CCL3 ELISA	R&D Systems	Cat#: DY450
Mouse TNFa ELISA	R&D Systems	Cat#: DY410
Mouse IL-6 ELISA	R&D Systems	Cat#: DY406
Live/Dead Fixable Aqua Dead Cell Stain Kit	Thermo Scientific	Cat#: L34966
Cyto-Fast Fix/Perm Buffer Set	BioLegend	Cat#: 426803
Experimental models: Organisms/strains		
Mouse: Mrgprb2−/−; C57BL/6J Mrgprb2 knockout	This lab	N/A
Mouse: Mrgprb2-Cre; C57BL/6J Cre-recombinase under Mrgprb2 promoter	This lab	N/A
Mouse: tdTomato (tdT); B6.Cg-Gt(ROSA)26Sor^tm14(CAG-tdTomato)Hze^/J	Jackson Labs	Jax: 007914; RRID: IMSR_JAX:007914
Mouse: C57BL/6-Tg(UBC-GFP)30Scha/J	Jackson Labs	Jax: 004353; RRID: IMSR_JAX:004353
Mouse: WT C57BL/6J	Jackson Labs	Jax: 000664; RRID: IMSR_JAC:000664
Oligonucleotides		
Primers targeting Mrgprb2: forward 5′-gtcacagaccagtttaacacttcc, rever 5′-cagccatagccaggttgagaa	This lab	N/A
Software and algorithms		
Prism	GraphPad Software	RRID: SCR_002798
FlowJo	BD	RRID: SCR_008520
ImageJ	NIH	RRID: SCR_003070
Other		
StemPro-34 SFM (1X)	Thermo Fisher	Cat#: 10639011
Isoflurane	Abbott Laboratories	Cat#: 26675-46-7
6-0 medium MCAO suture L910	Doccol Corporation	Cat#: 6023910PK10
RPMI 1640 Medium	Thermo Fisher	Cat#: 11875119
Percoll	Cytiva	Cat#: 17-0891-01

## Highlights

- Mrgprb2/MRGPRX2 is a key receptor that activates meningeal mast cells after stroke.
- Mrgprb2 regulates skull bone marrow neutrophil recruitment into the brain post-stroke.
- Mast cell proteases cleave semaphorin, mediating neutrophil infiltration into the brain.
- Inhibiting Mrgprb2 alleviates post-stroke brain inflammation and improves survival.
