# Supplementary figures and images for: A mast cell receptor mediates post-stroke brain inflammation via a dural-brain axis
Source: Cell. Author manuscript; Available in PMC 2025 Jul 31. (PMC12313293; doi:10.1016/j.cell.2025.06.045)

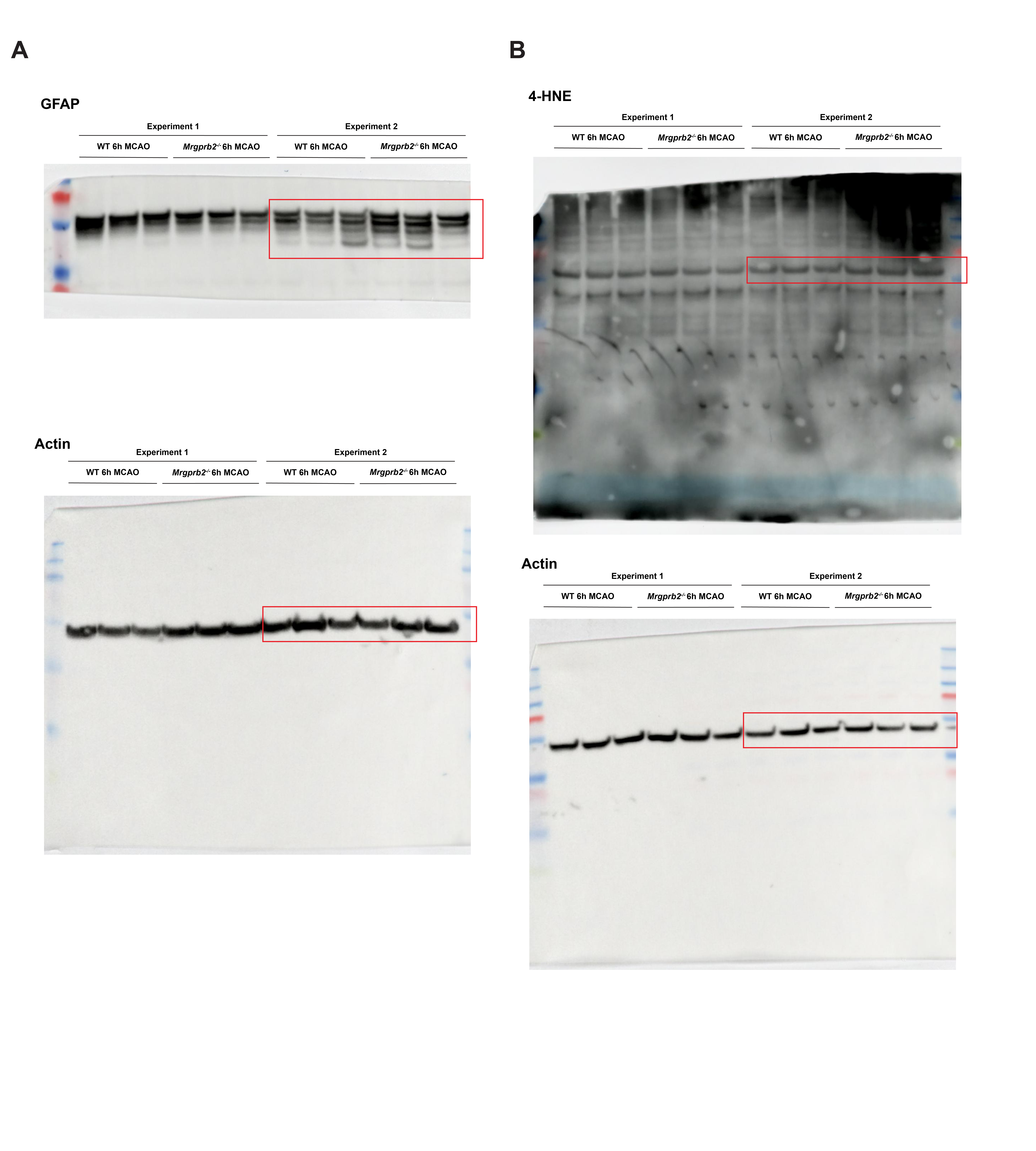

Supplement: Supplementary Data S1 — Data S1. Raw western blot images of GFAP and 4-HNE, related to Figure S3. [file NIHMS2094933-supplement-Supplementary_Data_S1.jpg]

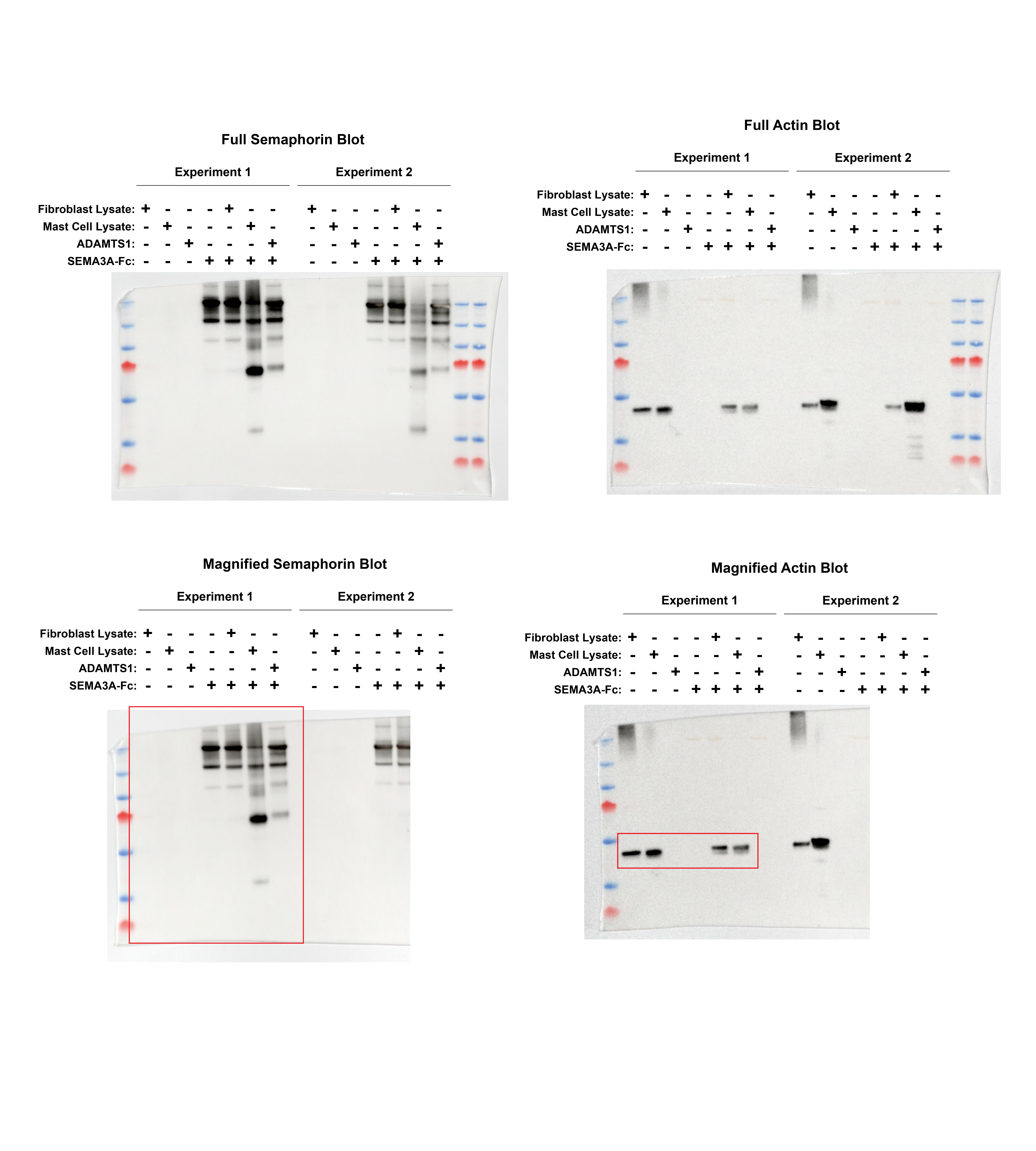

Supplement: Supplementary Data S2 — Data S2. Raw western blot images of semaphorin cleavage, related to Figure 4. [file NIHMS2094933-supplement-Supplementary_Data_S2.jpg]

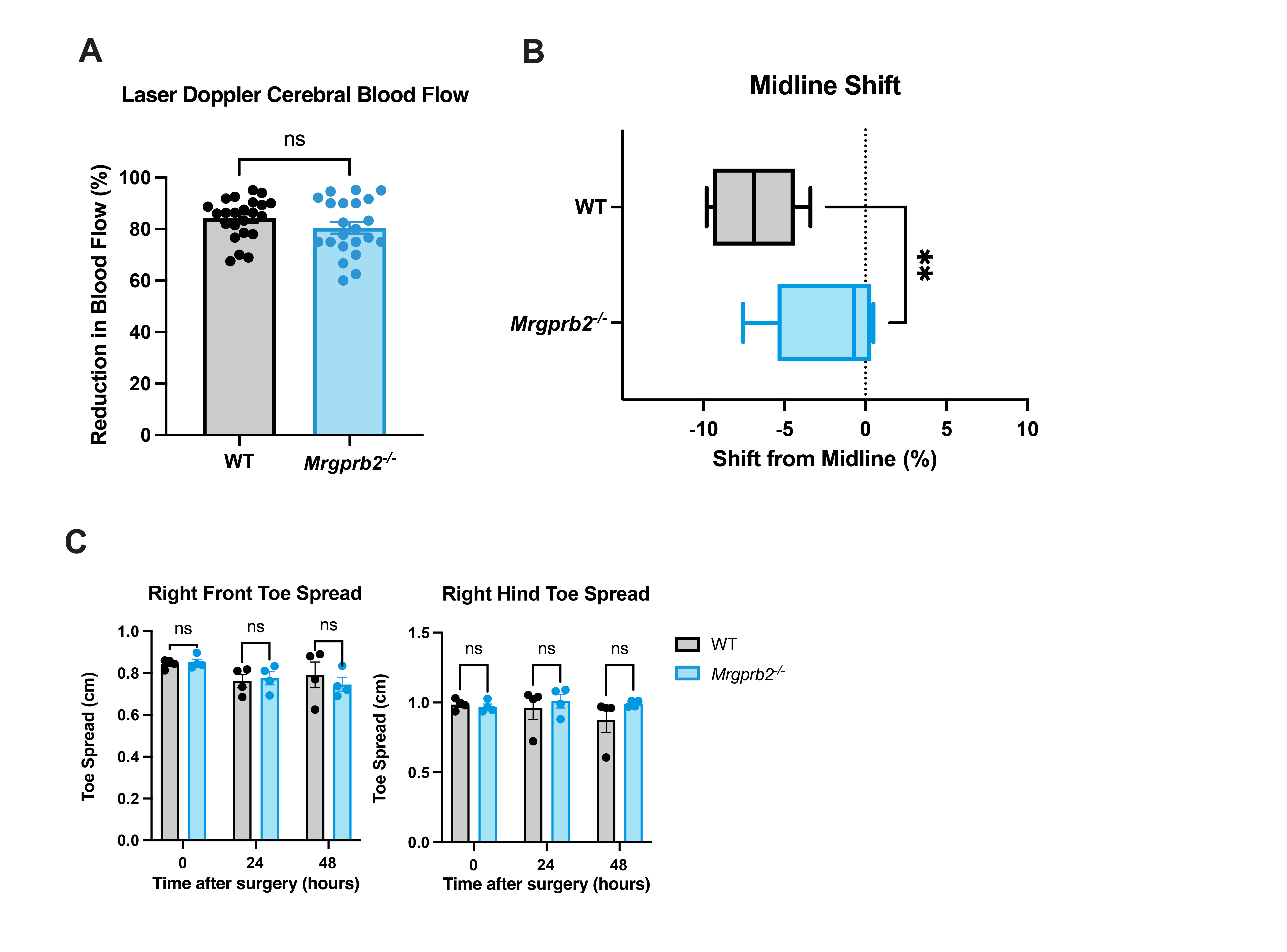

Supplement: Supplementary Figure S1 — Figure S1 related to Figure 1. Mrgprb2−/− mice exhibit equivalent blood flow reduction during tMCAO but have improved neurological outcomes. (A) Blood flow reduction during 40-minute occlusion time measured by laser doppler. Baseline blood flow measured before surgery is 100% (WT n=23, Mrgprb2−/− n=22). (B) Brain midline shift determined by MR imaging 48h after stroke. Negative shift indicates midline of the brain shifted away from the right (stroke) hemisphere into the left hemisphere. Whiskers indicate minimum and maximum values, and bold line depicts the mean (WT n=8, Mrgprb2−/− n=11). (C) left, Right front paw and right, right hind paw toe spread measured by Catwalk for WT/Mrgprb2−/− mice pre- and post-tMCAO (WT n=4, Mrgprb2−/− n=4). Statistical analyses: two-sided Student’s t-test (A), Mann-Whitney test (B), and two-way ANOVA with Sidak’s multiple comparisons test (C). Bar graphs indicate mean ± SEM. ns, not significant; **P < 0.01. [file NIHMS2094933-supplement-Supplementary_Figure_S1.jpg]

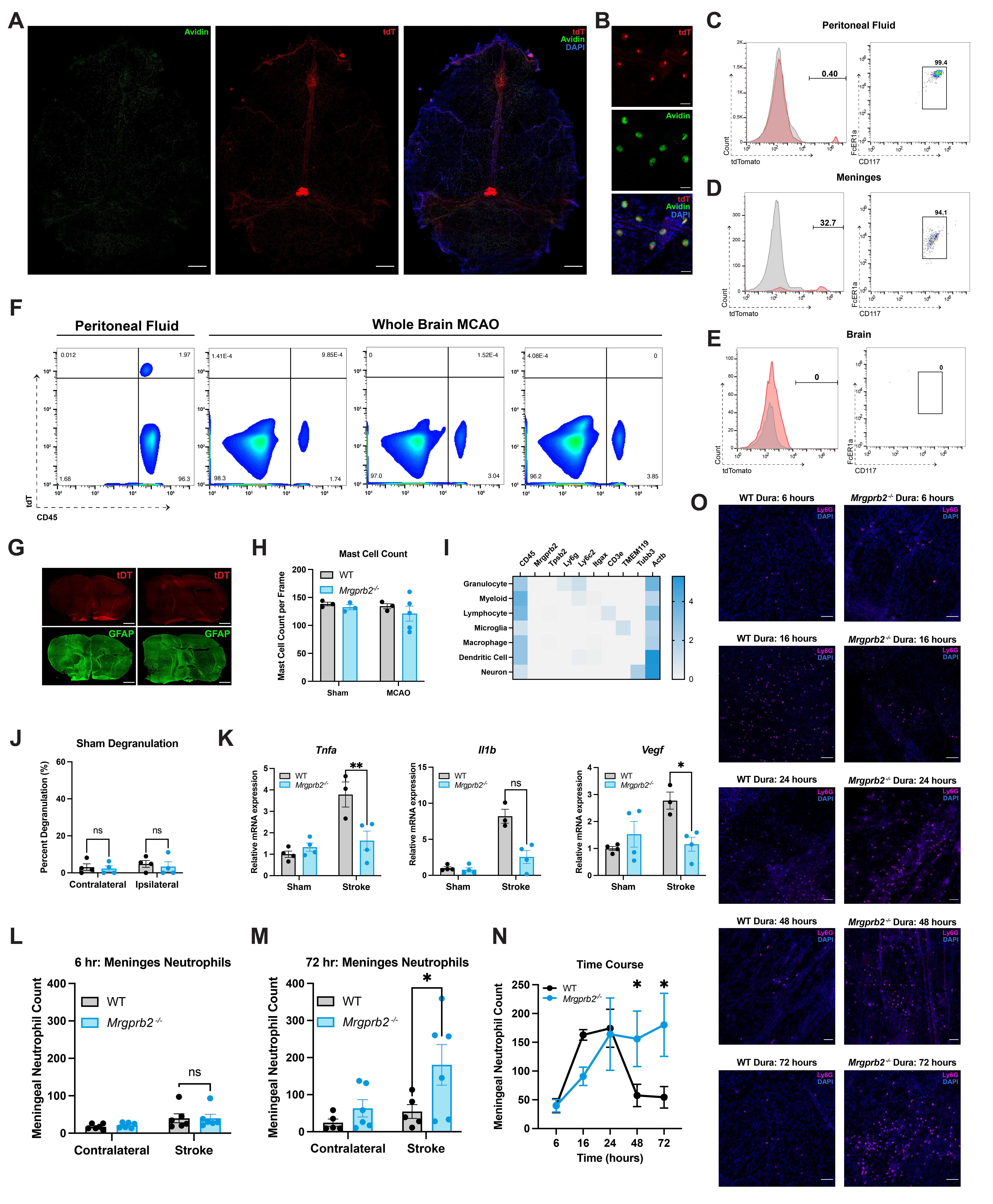

Supplement: Supplementary Figure S2 — Figure S2 related to Figure 2. Mrgprb2 shows 100% penetrance in mast cells of the meninges and Mrgprb2−/− mice show significant differences in neutrophil accumulation within the meninges after stroke. (A) Whole mount dura from Mrgprb2-Cre;tdT mice. left, Avidin signal identifies mast cells. middle, tdT signal correlates to Mrgprb2 expression. right, Avidin and tdT co-localize in every mast cell. DAPI is used to identify nuclei. Scale bar=500 μm. (B) Zoom of whole mount dura. Same colors as in A. Scale bar=25 μm. (C-E) (C) Peritoneal fluid, (D) dural meninges, and (E) whole brain from Cre− and Cre+ mice. Cre− mice do not express tdT and are used as negative control to properly gate each tissue for positive tdT signal. left, All CD45-positive immune cells from each tissue are plotted. Gray histogram is Cre− tissue, red histogram is Cre+ tissue. Number indicates frequency of Cre+ immune cells that are tdT positive. right, tdT positive cells gated for mast cells using CD117 and FcER1α. Number is frequency of tdT cells that are mast cells. (F) Flow cytometry of Mrgprb2-Cre;tdT peritoneal fluid, and whole brain 48h post-tMCAO. Each whole brain panel denotes a distinct mouse. Live cells gated for CD45 and tdT. Number in each quadrant indicates frequency of that population in all live cells. (G) Representative sections from anterior to posterior of one Mrgprb2-Cre;tdT mouse brain 48h after stroke. top, tdT fluorescence indicates Mrgprb2-expressing cells. No tdT positive cells are seen. bottom, GFAP indicates activated astrocytes that surround infarcted brain tissue. Scale bar=1000 μm. (H) Mast cell count in WT/Mrgprb2−/− mice in sham and tMCAO mice 48h after surgery, determined by avidin positive cells in the dura. Count is per frame using a 2.24mm2 viewing frame (WT sham n=3, Mrgprb2−/− sham n=3, WT tMCAO n=3, Mrgprb2−/− tMCAO n=5). (I) Average expression of mRNA using single cell analysis of the whole brain using the Brain Cell Data Viewer (https://www.braincelldata.org/s [file NIHMS2094933-supplement-Supplementary_Figure_S2.jpg]

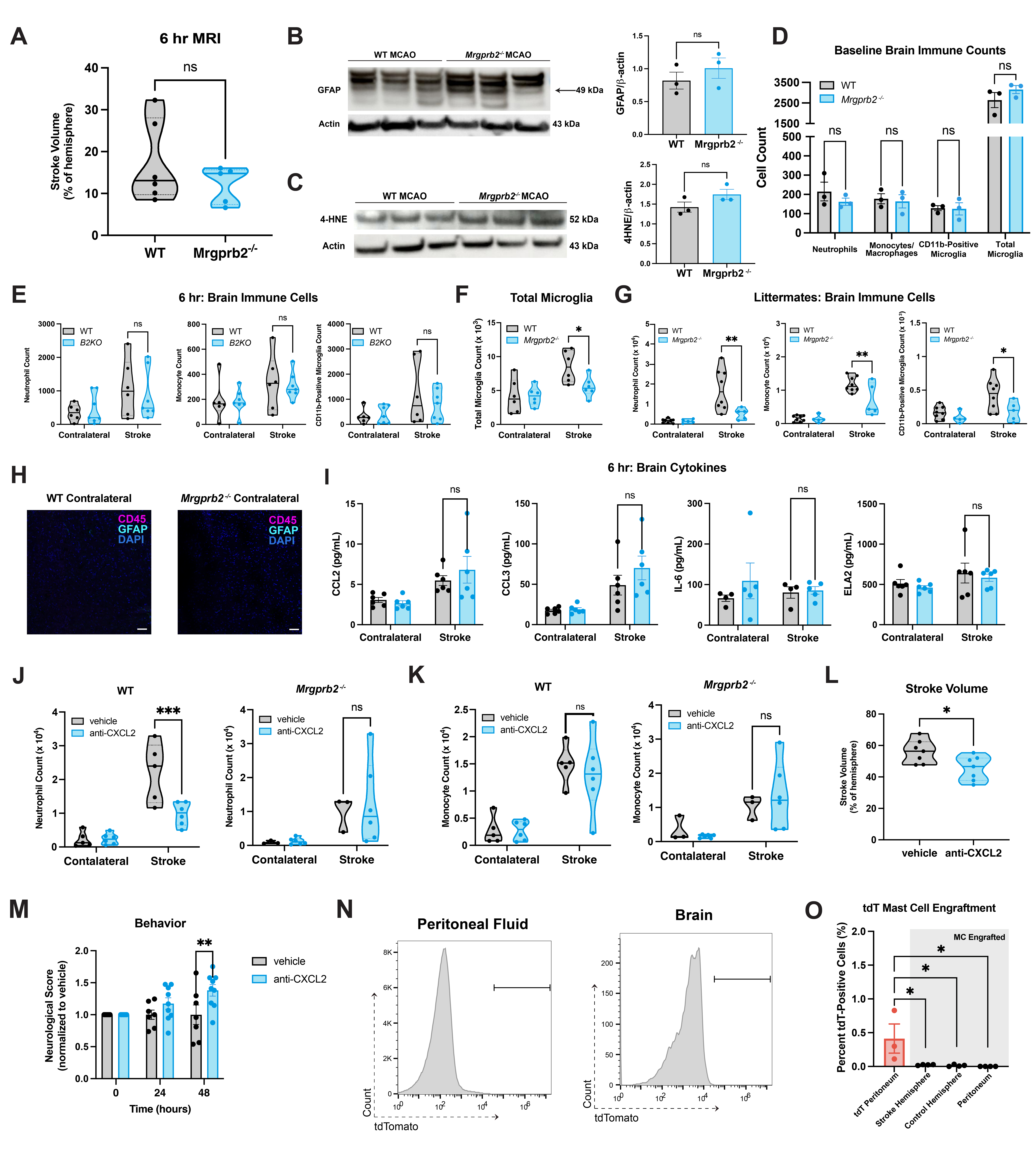

Supplement: Supplementary Figure S3 — Figure S3 related to Figure 3. WT/Mrgprb2−/− mice demonstrate similar ischemic injury immediately after stroke but show significant differences in neutrophil accumulation after stroke. (A) Quantification of stroke volume in WT/Mrgprb2−/− mice 6h post-tMCAO (WT n=6, Mrgprb2−/− n=5). (B-C) Western blot and quantification of (B) GFAP (main band at 49 kDa) and (C) 4-hydroxynonenal in WT/Mrgprb2−/− brains 6h post-tMCAO (WT n=3, Mrgprb2−/− n=3). (D) Flow cytometry of baseline WT/Mrgprb2−/− whole brains without tMCAO surgery. Cell counts of neutrophils, monocytes/macrophages, CD11b-positive microglia, and total microglia are denoted (WT n=3, Mrgprb2−/− n=3). (E) Absolute count of left, neutrophils, middle, monocytes/macrophages, and right, CD11b-positive microglia in contralateral and stroke brain hemispheres of WT/Mrgprb2−/− mice 6h post-tMCAO (WT n=6, Mrgprb2−/− n=7). (F) Absolute count of total microglia in contralateral and stroke brain hemispheres of WT/Mrgprb2−/− mice 48h post-tMCAO (WT n=6, Mrgprb2−/− n=6). (G) Absolute count of left, neutrophils, middle, monocytes/macrophages, and right, CD11b-positive microglia in contralateral and stroke brain hemispheres of WT/Mrgprb2−/− littermate mice (WT n=8, Mrgprb2−/− n=5). (H) Representative immunofluorescence images of left, WT, and right, Mrgprb2−/− contralateral brain hemispheres 48h post-tMCAO. CD45 denotes immune cells, GFAP counterstain delineates activated astrocytes, and DAPI identify nuclei. Scale bar=50 μm. (I) Protein expression measured by ELISA of various cytokines and chemokines in contralateral and stroke brain hemispheres of WT/Mrgprb2−/− mice 6h post-tMCAO (CCL2: WT n=6, Mrgprb2−/− n=6, CCL3: WT n=6, Mrgprb2−/− n=6, IL-6: WT n=4, Mrgprb2−/− n=5, ELA2: WT n=6, Mrgprb2−/− n=6). (J) Absolute count of neutrophils in the brain at 48h in left, WT and right, Mrgprb2−/− mice injected with vehicle or anti-CXCL2 antibody at 6h and 24h post-MCAO in the cisterna magna (WT vehicle n=5, WT anti-CXCL2 n=6, Mrgprb2−/− veh [file NIHMS2094933-supplement-Supplementary_Figure_S3.jpg]

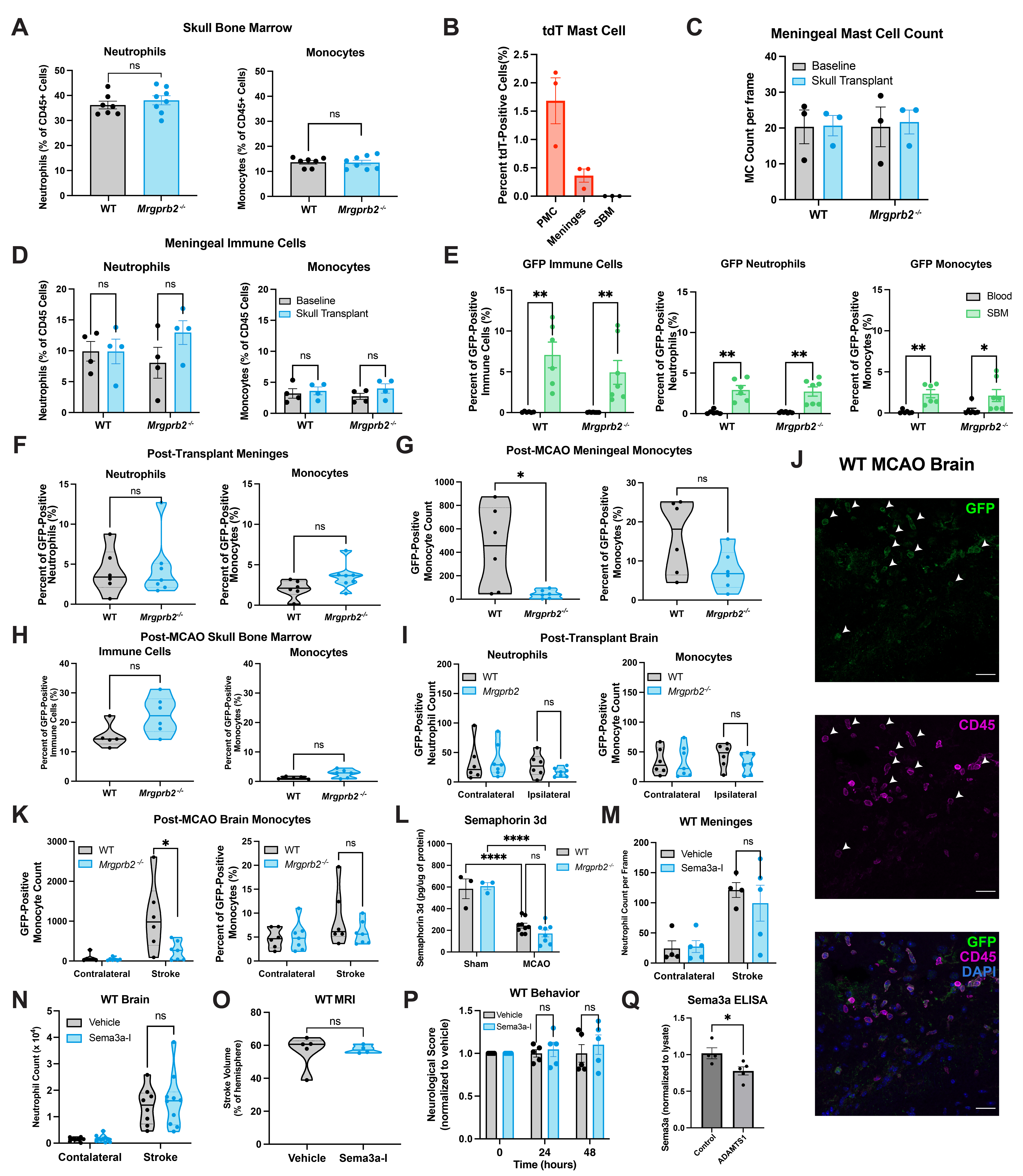

Supplement: Supplementary Figure S4 — Figure S4 related to Figure 4. GFP-positive cells predominate in the skull bone marrow after skull transplantation. (A) Percent of left, neutrophils and right, monocytes among all immune cells in the skull bone marrow in WT/Mrgprb2−/− mice (WT n=7, Mrgprb2−/− n=8). (B) Percent of Mrgprb2-tdT mast cells in all immune cells among peritoneal fluid, meninges, and skull bone marrow (SBM) (n=3 mice per tissue). (C) Mast cell count in WT/Mrgprb2−/− mice at baseline and 10 days after skull transplant determined by avidin positive cells in the dura. Count is per frame using a 0.408mm2 viewing frame (WT baseline/transplant n=3, Mrgprb2−/− baseline/transplant n=3). (D) Percent of left, neutrophils and right, monocytes among all immune cells in the meninges of WT/Mrgprb2−/− mice at baseline at 10 days after skull transplant (WT baseline n=4, Mrgprb2−/− baseline n=4, WT transplant n=4, Mrgprb2−/− transplant n=4). (E) left, Percent of GFP-positive immune cells, middle, GFP-positive neutrophils, and right, GFP-positive monocytes in the blood and SBM 10 days after skull transplant in WT/Mrgprb2−/− mice (WT n=6, Mrgprb2−/− n=7). (F) Percentage of left, GFP-positive neutrophils and right, GFP-positive monocytes in WT/Mrgprb2−/− meninges after skull transplant and 48h after sham surgery (WT n=6, Mrgprb2−/− n=7). (G). left, Absolute count of GFP-positive monocytes and right, percentage of total monocytes that are GFP-positive in dura of WT/Mrgprb2−/− mice 48h post-tMCAO (WT n=6, Mrgprb2−/− n=6). (H) Percentage of left, total immune cells and right, total monocytes that are GFP-positive in skull bone marrow of WT/Mrgprb2−/− mice 48h post-tMCAO (WT n=5, Mrgprb2−/− n=6). (I) Absolute count of GFP-positive left, neutrophils and right, monocytes in contralateral and ipsilateral brain hemispheres after skull transplant and 48h after sham surgery (WT n=6, Mrgprb2−/− n=7). (J) Representative immunofluorescence images of WT recipient right brain hemisphere 48h post-tMCAO. top, GFP denotes cells [file NIHMS2094933-supplement-Supplementary_Figure_S4.jpg]

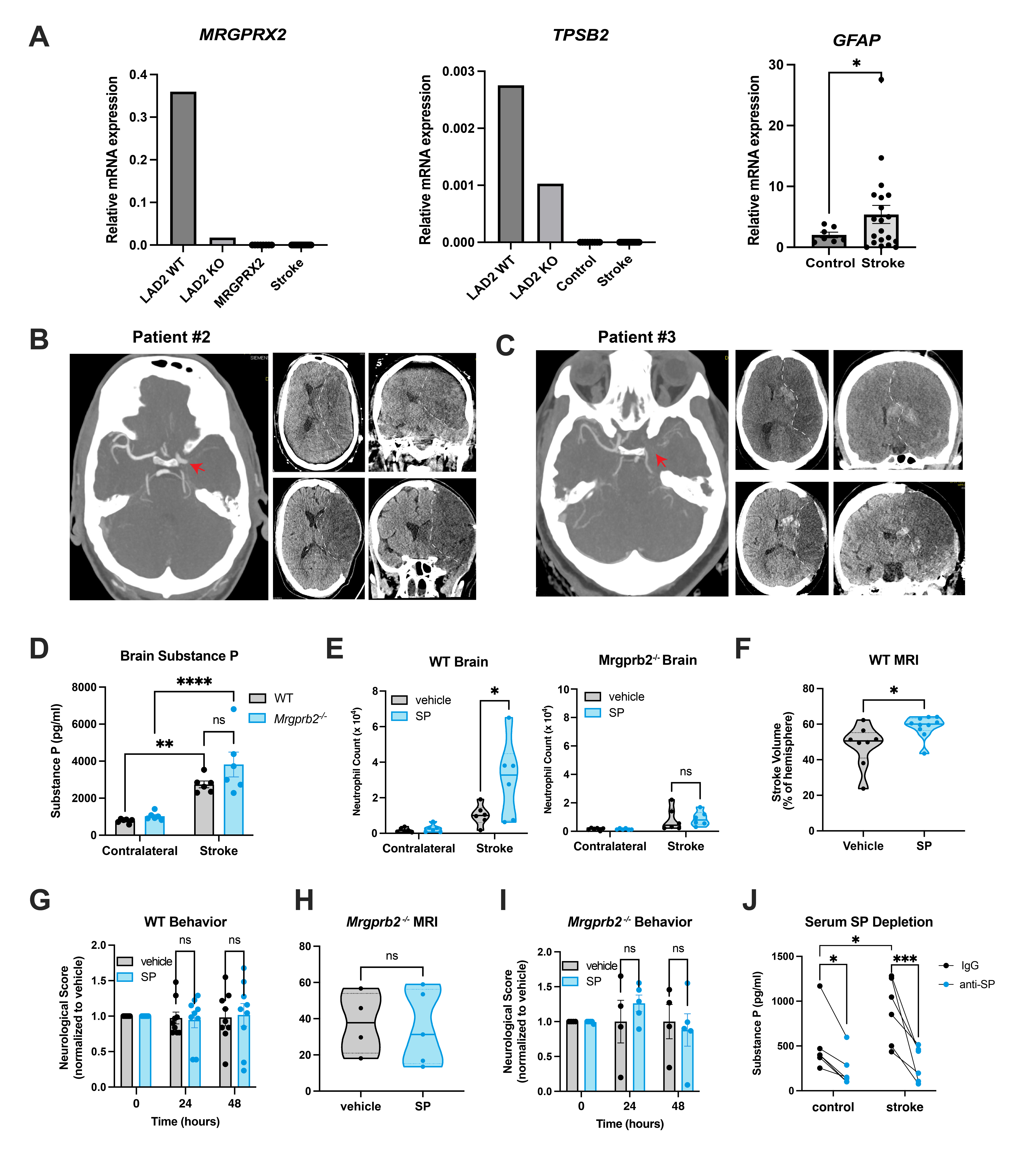

Supplement: Supplementary Figure S5 — Figure S5 related to Figure 5. Human stroke brain tissue does not express MRGPRX2, and Substance P is increased in the stroke mouse brain and human stroke serum. (A) Relative mRNA expression of MRPRX2 (left), tryptase (middle), and GFAP (right), in control and stroke patient brain tissue, normalized to β-actin expression. LAD2 WT and LAD2 KO cells serve as positive and negative controls for MRGPRX2 expression, and as positive controls for TPSB2 expression (MRGPRX2: control n=8, stroke n=24, TPSB2: control n=10, stroke n=26, GFAP control n=7, stroke n=20). (B) left, CT-angiogram of patient #2 (patient data in Table S2) presenting with left ICA termination occlusion. Arrow pointing at site of occlusion. right, top, Axial and coronal CT images of patient at time of presentation and bottom, axial and coronal CT images of patient after decompressive hemicraniectomy. Dotted lines denote infarcted region. (C) left, CT-angiogram of patient #3 (patient data in Table S2) presenting with left supraclinoid ICA occlusion. Arrow pointing at site of occlusion. right, top, Axial and coronal CT images of patient at time of presentation and bottom, axial and coronal CT images of patient after decompressive hemicraniectomy. Dotted lines denote infarcted region. (D) Quantification of substance P neuropeptide measured by ELISA in WT/Mrgprb2−/− contralateral and stroke brain hemispheres 48h post-tMCAO (WT n=6, Mrgprb2−/− n=6). (E) Absolute count of neutrophils at 48h in left, WT and right, Mrgprb2−/− injected with vehicle or SP at 6h and 24h post-MCAO in the cisterna magna (WT vehicle n=6, WT SP n=6, Mrgprb2−/− vehicle n=6, Mrgprb2−/− SP n=6). (F) Quantification of stroke volume in vehicle and SP treated WT mice 48h post-tMCAO (vehicle n=8, SP n=10). (G) Vehicle and SP treated WT mice neurological scores pre- and post-tMCAO. Scores normalized to vehicle-treated mice within cohorts (vehicle n=9, SP n=9). (H) Quantification of stroke volume in vehicle and SP treated Mrgprb2−/− mice 48h pos [file NIHMS2094933-supplement-Supplementary_Figure_S5.jpg]

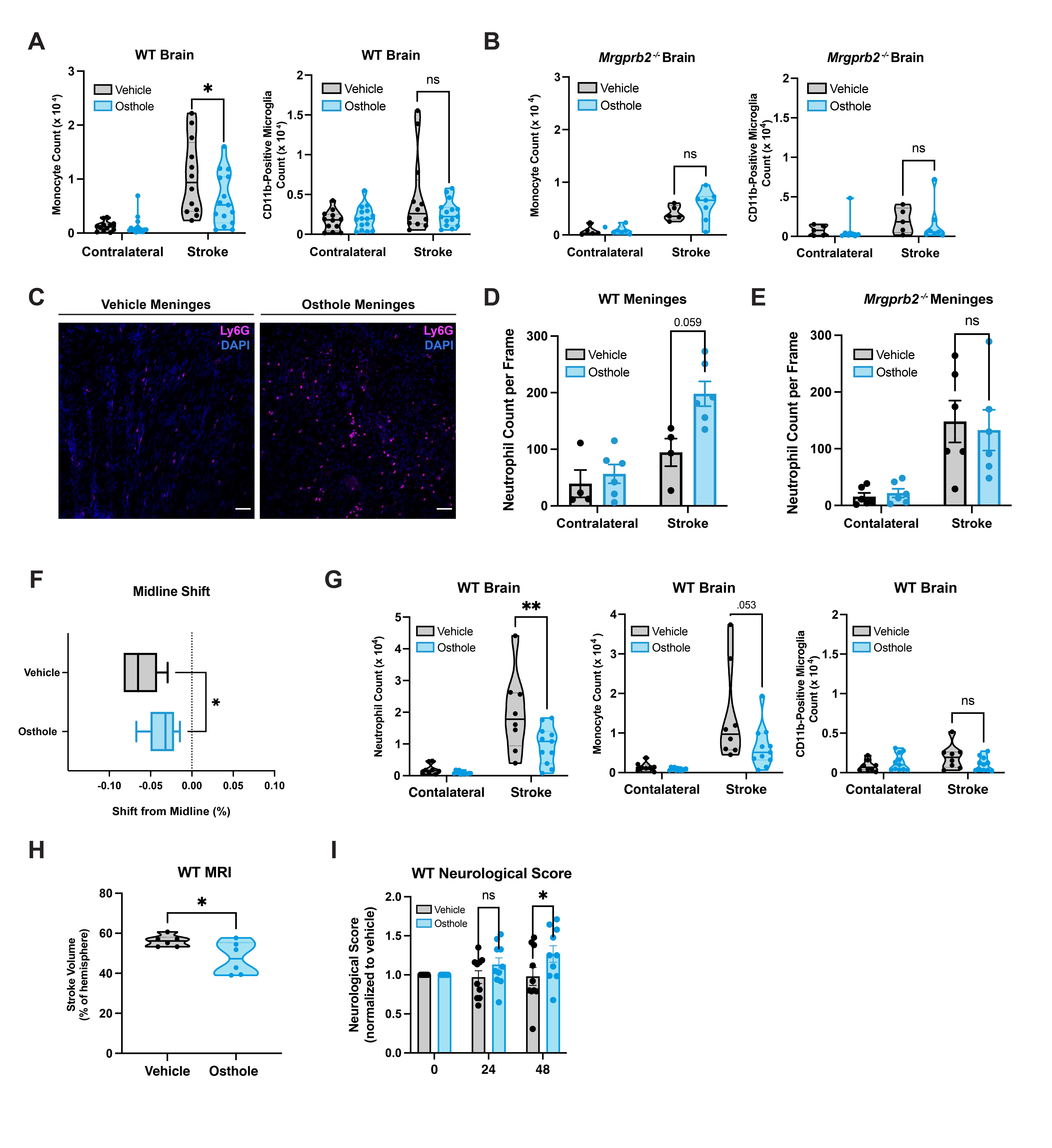

Supplement: Supplementary Figure S6 — Figure S6 related to Figure 6. Osthole reduces inflammation and injury in WT but not Mrgprb2−/− mice. (A) Absolute count of left, monocytes/macrophages and right, CD11b-positive microglia in contralateral and stroke brain hemispheres of WT vehicle and osthole treated mice 48h tMCAO (vehicle n=12, osthole n=15). (B) Absolute count of left, monocytes/macrophages and right, CD11b-positive microglia in contralateral and stroke brain hemispheres of Mrgprb2−/− vehicle and osthole treated mice 48h post-tMCAO (vehicle n=5, osthole n=7). (C) Representative immunofluorescence images of WT left, vehicle and right, osthole treated right hemispheric dura 48h post-tMCAO. Ly6G denotes neutrophils and DAPI identifies nuclei. Scale bar=50 μm. (D-E) Neutrophils in vehicle and osthole treated (D) WT and (E) Mrgprb2−/− dura 48h post-tMCAO (WT vehicle n=4, WT osthole n=6, Mrgprb2−/− vehicle n=6, Mrgprb2−/− osthole n=6). (F) Brain midline shift determined by MR imaging 48h after stroke. Negative shift indicates midline of the brain shifted away from the right (stroke) hemisphere into the left hemisphere. Whiskers indicate minimum and maximum values, and bold line depicts the mean (vehicle n=7, osthole n=7). (G) Absolute count of left, neutrophils, middle, monocytes/macrophages, and right, activated CD11b-positive microglia in contralateral and stroke brain hemispheres of cisterna magna vehicle and osthole treated WT mice 48h post-tMCAO (vehicle n=8, osthole n=11). (H) Quantification of stroke volume in cisterna magna vehicle and osthole treated WT mice 48h post-tMCAO (vehicle n=6, osthole n=6). (I) Cisterna magna vehicle and osthole treated WT mice neurological scores pre- and post-tMCAO. Scores normalized to vehicle-treated mice within cohorts (vehicle n=10, osthole n=10). Statistical analyses: two-way ANOVA with Sidak’s multiple comparisons test (A-B, D-E, G, and I) and two-sided Student’s t-test (F and H). Statistical test for A, B, D, and G (monocytes) was performed on log-transforme [file NIHMS2094933-supplement-Supplementary_Figure_S6.jpg]
