# Supplementary material for: A mast cell receptor mediates post-stroke brain inflammation via a dural-brain axis
Source: Cell. Author manuscript; Available in PMC 2025 Jul 31. (PMC12313293; doi:10.1016/j.cell.2025.06.045)
Supplement: Supplementary Tables S1-S3 — Table S1 Related to Figure 5: Postmortem brain patient characteristics used in qPCR analysis. Table S2 Related to Figure 5: Demographic and clinical presentation of ischemic stroke patients and controls used in human dura analyses. Table S3 related to Figure 5: Demographic and clinical presentation of ischemic stroke patients and controls used in human blood analyses. Data are presented as number of patients (% of total). [file NIHMS2094933-supplement-Supplementary_Tables_S1-S3.pdf]

	Age	Gender	Race	Infarct Age
Stroke	43	M	Black	Acute
	81	M	Hispanic	Acute
	47	M	Caucasian	Acute
	61	M	Hispanic	Acute
	61	M	Black	Acute
	71	M	Hispanic	Acute
	76	F	Hispanic	Acute
	95	M	Caucasian	Acute
	90	F	Black	Acute
	76	M	Caucasian	Acute
	66	M	Black	Acute
	77	M	Black	Acute
	61	M	Black	Subacute
	85	M	Black	Subacute
	94	F	Caucasian	Subacute
	83	F	Black	Subacute
	76	F	Hispanic	Subacute
	69	F	Black	Subacute
	53	F	Caucasian	Subacute
	67	F	Black	Late subacute
	66	F	Black	Late subacute
	67	F	Black	Chronic
	65	F	Black	Chronic
Control	68	M	NA	-
	66	M	White	-
	68	M	NA	-
	80	F	Hispanic	-
	64	M	Caucasian	-
	65	F	Black	-
	64	M	Black	-
	59	M	Black	-
	74	F	Asian	-
	77	F	Caucasian	-
	73	M	Hispanic	-
	61	M	Hispanic	-
	82	M	Asian	-

Table S1 related to Figure 5: Post mortem brain patient characteristics used in qPCR analysis.

	Stroke			Control		
**Patient**	1	2	3	1	2	3
**Age**	55	57	50	64	61	62
**Sex**	Female	Male	Female	Female	Female	Female
**Race**	Asian	White	Black	White	Black	Black
**Comorbidities**						
Hypertension	+	+	+	+	+	+
Hyperlipidemia	-	+	+	+	-	-
Chronic kidney disease	-	-	-	-	-	-
Coronary artery disease	-	-	-	-	-	-
Diabetes Mellitus	+	-	-	-	-	-
Atrial Fibrillation	+	-	-	+	-	-
**Smoking history**	-	-	-	+	NA	-
**Indication**						
Stroke (Occluded Vessel)	MCA	ICA	ICA	-	-	-
Emergent procedure	Decompressive hemicraniectomy					
Elective procedure	-	-	-	Elective Craniotomy		

Table S2 related to Figure 5: Demographic and clinical presentation of ischemic stroke patients and controls used in human dura analyses.

	Stroke	Control
**Patient**	17	11
**Age in years (SD)**	66.82 (19.06)	46.83 (15.32)
**Sex**		
Female	9 (52.9)	9 (81.8)
Male	8 (47.1)	2 (18.2)
**Race**		
White	9 (52.9)	9 (81.8)
Black	3 (17.6)	1 (9.1)
Asian	5 (29.4)	1 (9.1)
**Comorbidities**		
Hypertension	16 (94.1)	2 (18.2)
Hyperlipidemia	8 (47.1)	2 (18.2)
Chronic kidney disease	4 (23.5)	0 (0)
Coronary artery disease	6 (35.3)	2 (18.2)
Diabetes Mellitus	4 (23.5)	0 (0)
Atrial Fibrillation	3 (17.6)	1 (9.1)
**Smoking history**	7 (41.2)	4 (36.4)

Table S3 related to Figure 5: Demographic and clinical presentation of ischemic stroke patients and controls used in human blood analyses. Data are presented as number of patients (% of total).
